# Executive function and implications for the Preterm Behavioral Phenotype in very preterm children at age 9–10 years

**DOI:** 10.1186/s11689-026-09710-3

**Published:** 2026-05-28

**Authors:** Rachel E. Lean, Berenice Anaya, Lisa Gorham, Christopher D. Smyser, Cynthia E. Rogers

**Affiliations:** 1 Department of Psychiatry, Washington University School of Medicine, St. Louis, MO 63110 USA; 2 Department of Pediatrics, Washington University School of Medicine, St. Louis, MO 63110 USA; 3 Department of Radiology, Washington University School of Medicine, St. Louis, MO 63110 USA; 4 Department of Neurology, Washington University School of Medicine, St. Louis, MO 63110 USA; 5 Washington University School of Medicine, Campus Box 8514, St. Louis, MO 63110 USA

**Keywords:** Prematurity, White matter injury, Executive dysfunction, Psychopathology, Follow-up

## Abstract

**Background:**

Children born very preterm (VPT) have greater executive function (EF) challenges and internalizing, inattention, and social communication-interaction differences (the Preterm Behavioral Phenotype [PBP]) than full-term (FT) children. EF and PBP outcomes for VPT children with white matter injury (WMI) are less well understood. Furthermore, the extent that EF challenges serve as a neurocognitive mechanism for the PBP is unknown.

**Methods:**

As part of a longitudinal study, 123 VPT infants (≤ 30 weeks gestation) were recruited from a level-III neonatal intensive care unit and underwent developmental follow-up at ages 5 and 9–10 years. Forty-four VPT infants had high-grade WMI. Seventy-nine FT control children were also included. At the 5 and 9–10 year follow-up, children completed EF tasks tapping short-term/working memory, inhibitory control, and flexibility/shifting, which were combined into an EF composite at each timepoint. Parents and children also completed measures assessing children’s attention-deficit/ hyperactivity, internalizing/anxiety, and social communication-interaction outcomes to generate parent- and child-informant PBP composites at age 9–10 years. Serial mediation analysis examined EF at ages 5 and 9–10 years as serial mediators linking VPT birth and WMI with the PBP, adjusted for covariate factors.

**Results:**

VPT and WMI children demonstrated lower EF abilities at both timepoints compared to FT children (*p*≤.02). VPT and WMI children obtained higher child-informant PBP composite scores (*p*<.001) as well as parent- and child-informant social communication-interaction and ADHD-inattentive problem ratings (*p*≤.03) at age 9–10 years. Lower EF at both timepoints correlated with higher parent- and child-informant PBP ratings (*r* -0.2 to -0.45, *p*<.05). Serial mediation analysis showed that EF development from age 5 to 9–10 years (proportion mediated 3–5%) as well as EF at age 9–10 years (proportion mediated 16–43%) partially mediated associations linking VPT birth and WMI with PBP outcomes.

**Conclusions:**

VPT and WMI children had greater EF and PBP challenges compared to FT children, with WMI children at greatest risk. Disrupted EF development, at least in part, contributed to the PBP. Findings suggest that VPT and WMI children are in need of early and ongoing EF supports, and that EF training interventions may improve mental health outcomes in this population.

**Supplementary Information:**

The online version contains supplementary material available at 10.1186/s11689-026-09710-3.

In the United States, one in ten infants are born preterm (< 37 weeks gestational age, GA) [1]. Although major advances in antenatal monitoring and neonatal care have led to increased survival rates for infants born very preterm (VPT, < 32 weeks GA) [2, 3], there has been no corresponding improvement in longer-term neurodevelopmental outcomes [3, 4]. For example, over four decades of research has consistently shown that VPT children demonstrate poorer cognitive development compared to full-term (FT) children [5, 6]. A large meta-analysis reported that 8 to 15% of VPT children have severe cognitive impairment (full scale intelligence quotient, FSIQ < 70), with rates as high as 34% in individual studies [7]. This prevalence represents at least a four-fold increase in severe cognitive impairment compared to the general pediatric population [8, 9]. VPT infants with perinatal high-grade white matter injury (WMI), including grade III/IV intraventricular hemorrhage (IVH), post-hemorrhagic hydrocephalus (PHH), and cystic periventricular leukomalacia (cPVL), are a particularly vulnerable group. Up to half of VPT children with WMI have been identified has having cognitive delay as early as age 2 years [10–14]. Given the large and well-defined literature documenting major cognitive impairments among VPT children, more recent follow-up studies have shifted towards understanding challenges in nuanced areas of cognition, including attention and executive function (EF).

EF is a higher-order aspect of cognition that allows an individual to engage in effortful goal-directed behavior by coordinating and maintaining a set of inter-related lower-order cognitive processes including working memory, inhibitory control, and shifting/flexibility [15]. Findings from multiple follow-up studies indicate that VPT children obtain lower scores on standardized and experimental measures of EF and higher parent-report ratings on behavioral measures of executive dysfunction compared to FT children [16–18], with moderate-to-large effect sizes often reported [17, 19–21]. Furthermore, VPT children demonstrate pronounced difficulties on higher-order planning/organization, abstract reasoning, and problem solving tasks that place greater demands on the executive system [22, 23]. Importantly, associations between preterm birth and adverse EF outcomes persist after accounting for a range of confounding factors including exposure to sociodemographic disadvantage and maternal psychopathology, as well as lower general cognitive ability [4, 20, 21, 24].

To date, most previous research documenting the EF outcomes of VPT children has been cross-sectional [16–18]. Only a handful of studies have examined the longitudinal development of EF in VPT children, with mixed findings [24–28]. For example, Lee et al. found that VPT children performed less well on measures of planning and inhibitory control at age 6 years than FT children, but that VPT and FT children had similar EF abilities by ages 8 and 10 years [26]. These findings potentially suggest that VPT children demonstrate early delays in EF development that resolve by middle-to-late childhood. However, Lee et al. focused on a small cohort of preterm children without neurodevelopmental impairments who may be less likely to demonstrate longer-term EF challenges than other groups of VPT children, including those with neurological abnormalities identified as early as the neonatal period [24, 29]. Just two studies have examined intra-individual changes in EF in VPT children and FT children from school-age to adolescence. Both Burnett et al. [25] and Everts et al. [28] found that VPT children showed more rapid gains in EF development over time compared to FT children. However, Burnett et al. [25] also found that the VPT group still obtained lower EF scores than the FT group at age 17 years, suggesting that despite gains in EF development, VPT adolescents continued to lag behind their FT peers. While this small collection of longitudinal studies provides important insight on EF development in VPT children, EF development in VPT children with high-grade WMI remains unclear. Previous longitudinal studies of EF in childhood and adolescence have either excluded VPT children with brain injury [26, 28], combined brain injury status with non-neurological factors as part of a clinical risk composite score [27], included few VPT children with brain injury [25], or conducted blunt categorical analysis of pass/failure rates on EF tasks in VPT children with moderate/severe white matter abnormalities assessed with qualitative ratings [24]. These methodological issues have left the EF outcomes of WMI children, who are in greatest need of ongoing developmental support, poorly understood.

In addition to poorer EF outcomes, there is mounting evidence that VPT children experience higher levels of co-occurring internalizing/anxiety symptoms, attention problems, and social communication-interaction differences; a constellation of symptoms known as the Preterm Behavioral Phenotype (PBP) [30]. In the wider literature, executive dysfunction is a well-established transdiagnostic risk factor for attention-deficit/hyperactivity disorder (ADHD), mood/affective disorders, and Autism [31–34]. These associations reflect the extent that EF acts as a central top-down system that regulates impulse control, inhibits maladaptive or goal-irrelevant thoughts and behaviors, and supports the capacity to flexibly adjust to the changing demands of the physical and social environment for healthy socioemotional functioning [33, 35, 36]. Indeed, executive dysfunction has been found to play a unique role in increasing psychiatric symptoms over time. Children who perform less well on EF tasks show increased psychiatric symptoms at later follow-up [32, 37, 38], even after controlling for baseline socioemotional or psychiatric symptoms [39, 40]. However, relatively little is known about the extent that executive dysfunction relates to the PBP in VPT or WMI children. Existing studies in VPT children have examined EF in relation to a single domain of the PBP [41–44] or global measures of socioemotional function that incorporate internalizing, externalizing, and peer relationship difficulties [45]. For example, higher parent-report ratings on behavioral measures of executive dysfunction correlate with more severe ADHD symptoms in preschool-aged VPT children [44]. Regarding links between EF and social communication-interaction differences, both parent-report and task-based assessments of executive dysfunction have been consistently associated with challenges in social competency, social cognition, and emotion understanding in VPT children and extremely preterm children (EPT, born < 28 weeks GA) [41–43]. Cross-sectional mediation analysis has also shown that cognitive control, a higher-order aspect of cognition that overlaps with EF, accounts for the association between VPT birth and social challenges in adolescence, over and above other key social-affective processes like emotion recognition [46]. These collective findings align with conceptual models that emphasize EF as a critical neurocognitive mechanism that supports the top-down regulation of cognition and behavior, impairments in which drive psychopathology risk [33, 35, 36]. However, existing findings from VPT follow-up studies are largely based on cross-sectional associations [41–46] and thus the extent that early and persisting EF challenges contribute to the PBP in VPT and WMI children remains unclear.

To address these important research and clinical gaps, the current study draws data from a longitudinal cohort of WMI, VPT, and FT children assessed from birth to age 9–10 years. The current study had three key objectives. First, we compared the EF outcomes of WMI, VPT, and FT children in early and middle childhood. Second, we examined the stability of EF from early to middle childhood, and the extent that exposure to a range of socioenvironmental and maternal psychosocial factors in early childhood [21] related to change in EF, using residualized-change models. Third and last, serial mediation analysis was used to delineate the role of EF development across childhood as a potential mechanism linking VPT birth and WMI with subsequent PBP outcomes at age 9–10 years. We hypothesized that (a) VPT and WMI children would show greater EF and PBP challenges relative to FT children, with WMI children at greatest risk of poor outcomes, (b) individual differences in early childhood EF would relate to subsequent EF independent of key socioenvironmental and maternal psychosocial covariate factors that would also predict longer-term EF outcomes, and (c) poorer EF development across childhood would serve as a mediator linking VPT birth and WMI with PBP outcomes at age 9–10 years after accounting for covariate factors known to be implicated in executive dysfunction and developmental psychopathology.

## Methods

### Sample

The current study includes 204 children participating in a prospective longitudinal study investigating the neurodevelopmental and psychiatric outcomes of VPT children at Washington University School of Medicine (see Table 1 for sample characteristics). Excluding VPT participants who were deceased (*n* = 24) or withdrawn (*n* = 17), 183 infants born VPT (≤ 30 weeks GA) were recruited from the St. Louis Children’s Hospital Level-III neonatal intensive care unit (NICU), of which 123 completed the age 9–10 year follow-up (retention rate 67%). There were no differences in gestational age (*p* = .81), birthweight (*p* = .54), or neighborhood Area Deprivation Index percentiles at birth (*p* = .51) between VPT infants retained or not retained to the 9–10 year follow-up. A subset of VPT infants were identified as having perinatal high-grade WMI (*n* = 44) based upon clinical review of neonatal structural magnetic resonance imaging (MRI) scans and cranial ultrasound. WMI included Papile grade III/IV IVH [47], PHH (IVH requiring neurosurgical treatment for hydrocephalus based upon frontal–occipital ratio ≥ 0.55, progressive increase in occipitofrontal circumference, splaying of the sagittal suture ≥ 2 mm in the mid-parietal region, and palpation of the anterior fontanelle above the level of the surrounding bone) [48], or cPVL. Consistent with our prior work [10, 49], WMI infants with grade III/IV IVH (*n* = 26), PHH (*n* = 10), and cPVL (*n* = 8) were analyzed as a single group. A group of FT control children (*n* = 81) were recruited via two mechanisms; 56 FT children were identified as infants from a contemporaneous study at an adjoining obstetric service and 25 FT control children were recruited from the local community at age 5 years [21]. Exclusion criteria for all participants included parent unable to give informed consent, infant chromosomal/congenital abnormality, and suspected/proven congenital infection. Additional exclusion criteria for the FT group included maternal prenatal positive urine drug screen, neonatal acidosis (pH < 7.20) on umbilical cord or arterial blood gas in the hour of life, and brain abnormality on structural MRI. As part of the larger study, structural MRI scans obtained at the 9–10 year follow-up were also clinically reviewed by a neuroradiologist for the presence of brain abnormalities. At the time of the analysis, two FT children were being reviewed for possible brain abnormalities and were excluded from the current study.

**Table 1:** Background characteristics of white matter injury (WMI), very preterm (VPT), and full term (FT) children (*n* = 202)

m ± SD or % (*n*)	WMI **(*n*** **= 44)**	VPT **(*n*** **= 79)**	FT **(*n*** **= 79)**	F/t/x^2^	*p*
Infant clinical characteristics					
Gestational age (weeks)^a^	25.52 ± 1.75	26.33 ± 1.62	39.23 ± 1.11	2263.38	< 0.001
Birthweight (grams)^a^	884.66 ± 207.14	914.60 ± 238.93	3361.77 ± 503.01	820.61	< 0.001
Sex assigned at birth					
Male	63.6 (28)	51.9 (41)	45.6 (36)	3.70	0.16
Female	36.3 (16)	48.1 (38)	54.4 (43)		
Infant medical risk index	2.80 (1.92)	1.81 (1.85)	-	2.67	0.009
Oxygen at 36 weeks	79.5 (35)	51.4 (36)	-	9.09	0.003
Patent ductus arteriosus	20.5 (9)	40.0 (28)	-	4.71	0.04
Necrotizing enterocolitis	25.0 (11)	5.7 (4)	-	8.79	0.004
Sepsis	45.5 (20)	25.7 (18)	-	4.74	0.04
Retinopathy of prematurity	25.0 (11)	14.3 (10)	-	2.06	0.21
Received postnatal steroids	15.9 (7)	12.9 (9)	-	0.21	0.78
Did not receive antenatal steroids	31.8 (14)	9.5 (6)	-	8.47	0.005
Intrauterine growth restriction	2.3 (1)	6.5 (4)	-	1.00	0.40
Upper quartile for total parenteral nutrition	29.5 (13)	20.6 (13)	-	1.12	0.36
SDS decrease more than 3SD from birth to discharge	4.5 (2)	1.4 (1)	-	1.02	0.56
Developmental characteristics					
*Infant socioemotional problems at age 2 years*					
Internalizing symptoms	51.18 ± 10.11	48.54 ± 10.03	50.52 ± 10.83^b^	0.80	0.45
Externalizing symptoms	55.03 ± 10.20	53.61 ± 12.36	55.68 ± 15.70^b^	0.28	0.76
Dysregulation problems	55.26 ± 15.08	48.68 ± 12.81	46.55 ± 10.19^b^	3.31	0.04
Social competency skills	38.41 ± 14.71	41.69 ± 13.99	50.50 ± 12.60^b^	5.17	0.007
*General cognitive ability*					
FSIQ at age 5 years	79.86 ± 12.57	90.00 ± 14.09	98.67 ± 16.90	14.11	< 0.001
FSIQ < 70 at age 5 years	21.4 (6)	4.7 (3)	3.3 (1)	10.62	0.005
FSIQ at age 9–10 years	75.88 ± 16.59	85.99 ± 15.02	93.39 ± 19.51	13.88	< 0.001
FSIQ < 70 at age 9–10 years	35.0 (14)	8.9 (7)	8.9 (7)	17.96	< 0.001
Social and family characteristics at the 9–10 year follow-up					
Social disadvantage composite score	0.09 ± 0.84	-0.07 ± 1.00	0.02 ± 1.08	0.39	0.68
Family income-to-needs ratio^c^	2.29 ± 1.70	2.73 ± 2.25	2.70 ± 2.29	0.66	0.52
Area Deprivation Index percentile^c^	67.70 ± 24.24	65.81 ± 25.55	67.67 ± 29.96	0.12	0.89
Maternal socio-demographic stressor index^c^	1.77 ± 1.24	1.61 ± 1.32	1.82 ± 1.32	0.56	0.57
* Young mother at delivery (< 18 years)* ^d^	6.8 (3)	5.1 (4)	7.6 (5)	0.43	0.81
* No High School Qualification* ^d^	11.4 (5)	5.3 (4)	7.7 (6)	1.48	0.48
* Single parent household* ^d^	52.3 (23)	51.3 (39)	52.6 (41)	0.03	0.99
* Race Black or African American (proxy for racial discrimination)* ^d^	45.5 (20)	49.4 (39)	67.1 (53)	7.30	0.03
* Public health insurance* ^d^	61.4 (27)	50.0 (38)	47.4 (37)	2.30	0.32
Maternal depression symptoms	5.11 ± 6.72	5.08 ± 5.84	6.92 ± 7.65	1.71	0.18
Maternal anxiety symptoms	26.66 ± 7.65	28.81 ± 8.24	32.79 ± 11.18	6.41	0.002
Maternal social support (satisfaction)	5.60 ± 0.93	5.42 ± 1.25	5.35 ± 0.99	0.77	0.47
Parent stress index percentile	38.68 ± 34.34	34.20 ± 29.86	40.33 ± 33.54	0.70	0.50
Stressful/traumatic events percentile	44.55 ± 31.16	50.25 ± 33.08	55.79 ± 34.36	1.59	0.21
Maternal FSIQ	95.04 ± 13.9	96.59 ± 10.59	98.84 ± 15.26	0.78	0.46

^a^ ANOVA with Welch correction for unequal variances

^b^ Data reported for infant-recruited FT children assessed at age 2 years

^c^ Factors included in the social disadvantage composite

^d^ Factors included in the socio-demographic stressor index

### Procedures

At chronological ages 5 and 9–10 years, children completed a comprehensive neurodevelopmental evaluation involving cognitive and socioemotional assessments (see Table S1 for a summary of key measures). Children completed a battery of developmentally-appropriate tasks assessing short-term or working memory, inhibitory control, and flexibility/shifting skills. At the 9–10 follow-up, children additionally completed task-based assessments of social perception (affect recognition, theory of mind) and a self-report measure of socioemotional outcomes. The child’s primary caregiver completed questionnaires to obtain information about family socioenvironmental circumstances, parent mental health and wellbeing, and child socioemotional outcomes. Primary caregivers provided informed written consent. Children provided verbal assent at age 9–10 years. All study procedures were approved in accordance with the Declaration of Helsinki by the Washington University Institutional Review Board.

### Measures

#### Executive Function (EF)

At the 5 year follow-up, short-term memory was assessed using the Digits Forward subtest of the Differential Ability Scale [50], and inhibitory control and shifting/flexibility was assessed using The Shape School task [51]. These tasks have been used previously in the larger study to describe the EF outcomes of VPT and FT children, but not WMI children, at age 5 years [21]. At the 9–10 year follow-up, children completed the age-normed NIH Toolbox Cognitive Battery [52]. Key tasks included List Sorting Working Memory, Flanker Inhibitory Control and Attention, and Dimensional Card Sorting. We note that while our task batteries assessed different aspects of memory abilities at the 5 and 9–10 year timepoints, we broadly refer to these constructs using the collective term short-term/working memory in longitudinal analyses.

Tasks at each timepoint were reduced into a global EF composite score using Principal Component Analysis (PCA) [21]. Composite scores were used because EF shows a unitary structure in childhood [53, 54] and because composite scores capture shared variance across EF tasks thereby improving the reliability of EF measurement [55, 56]. In the current study cohort, EF tasks loaded on to a single common component at both the 5 year (54% variance explained, factor loadings ≥ 0.70) and the 9–10 year (67% variance explained, factor loadings ≥ 0.80) timepoints. Global EF at age 5 years was positively correlated with global EF at age 9–10 years (*r* = .37, *p* < .001, see Table S1 for domain-specific correlations).

#### Preterm Behavioral Phenotype (PBP)

PBP outcomes were assessed at age 9–10 years using a range of validated questionnaires and tasks. Parent-report measures for each PBP domain (see Table S1) included the Child Behavior Checklist/6–18 [57], Screen for Child Anxiety and Related Emotional Disorders [58], Social-Responsiveness Scale-2 [59], and The Conners-3rd Edition [60]. Child-informant measures included the self-report Brief Problem Monitoring-Youth [61] and task-based measures of social perception from the Developmental Neuropsychological Assessment-2 [62]. Parent- and child-informant PBP composite scores were generated using PCA. Parent-informant measures loaded onto a common parent-informant PBP component (62% variance explained, factor loadings ≥ 0.73). Although child-informant measures loaded onto two components, all child measures demonstrated stronger loadings on the first component than on the second component. Therefore, the first child-informant component was selected for analysis in the current study (51% variance explained, factor loadings ≥ 0.66). Parent- and child-informant PBP composite scores were positively correlated (*r* = .37, *p* < .001, see Table S1 for domain-specific correlations).

### Covariate factors of interest

#### Infant medical risk index

As described in our prior publications [21, 63], gestational age, birthweight, sex assigned at birth (male = 1, female = 2), race and ethnicity, and neonatal clinical morbidities were obtained from infant medical records. For VPT and WMI infants, the infant medical risk index was based on the presence (= 1) or absence (= 0) of ten clinical morbidities during the NICU stay (index range 0–10): intrauterine growth restriction, oxygen supplementation at 36 weeks, did not receive antenatal steroids, received postnatal dexamethasone, necrotizing enterocolitis, confirmed sepsis, patent ductus arteriosus, retinopathy of prematurity, ≥ 3 standard deviation decrease in weight-for-height/length by term-equivalent age, and > 75th percentile for total parenteral nutrition [21, 63].

#### Infant socioemotional problems at age 2 years

To account for early socioemotional problems that have been implicated in the development of psychiatric disorders, the parent-report Infant and Toddler Social and Emotional Assessment (ITSEA) [64] provided measures of externalizing symptoms, internalizing symptoms, dysregulation, and differences in social-competency at age 2 years. The ITSEA is age- and sex-normed and is corrected for prematurity up to chronological age 2 years.

#### General cognitive ability

Children’s general cognitive ability was assessed using the Weschler Preschool and Primary Scale of Intelligence-3rd Edition (WPPSI-III) [65] at age 5 years, and using the four-subtest Weschler Abbreviated Scale of Intelligence-2nd Edition (WASI-II) [66] at age 9–10 years. The WPPSI-III and WASI-II provide age-normed FSIQ scores encompassing fluid and crystallized intelligence. Severe cognitive impairment was defined as FSIQ < 70. Maternal cognitive ability was assessed using the brief Weschler Test of Adult Reading (WTAR) [67]. The WTAR is co-normed with the Weschler Adult Intelligence Scale-3rd Edition (WAIS-III). WTAR-predicted WAIS-III FSIQ scores, normed by maternal age, education level, and race, were used in analyses.

#### Socioenvironmental factors

Social and family background information was obtained at the 5 and 9–10 year follow-up assessments. Maternal sociodemographic factors, household income, number of people in the home, and home address zip codes were collected from a study specific social background questionnaire. As described in our prior works [21, 63], this information was used to generate a maternal sociodemographic stressor index (see Table 1), family Income-to-Needs Ratio, and neighborhood Area Deprivation Index percentile [68], respectively. As social disadvantage measures were inter-correlated (*r* range 0.56 to 0.66, all *p*<.001), a multidimensional social disadvantage composite score was generated for each time point using PCA [21, 63]. Measures of maternal mental health and wellbeing collected at each follow-up included the Beck Depression Inventory-II [69], State Trait Anxiety Inventory [70], Parent Stress Index (PSI) [71], Social Support Questionnaire [72], and PSI Life Events [71]. Maternal psychosocial measures were examined individually to identify specific risk domains that may be related to children’s EF outcomes. For parsimony in multivariable serial mediation analyses, a summary maternal psychosocial distress index was used as a covariate. As described in our prior work [23, 73], scores on each psychosocial measure were categorized into risk (i.e., score in the clinical range = 1) or no risk (i.e., score in the normal range = 0) and summed to create a maternal psychosocial stress index (index range 0–5).

### Data analysis plan

**Fig. 1:**
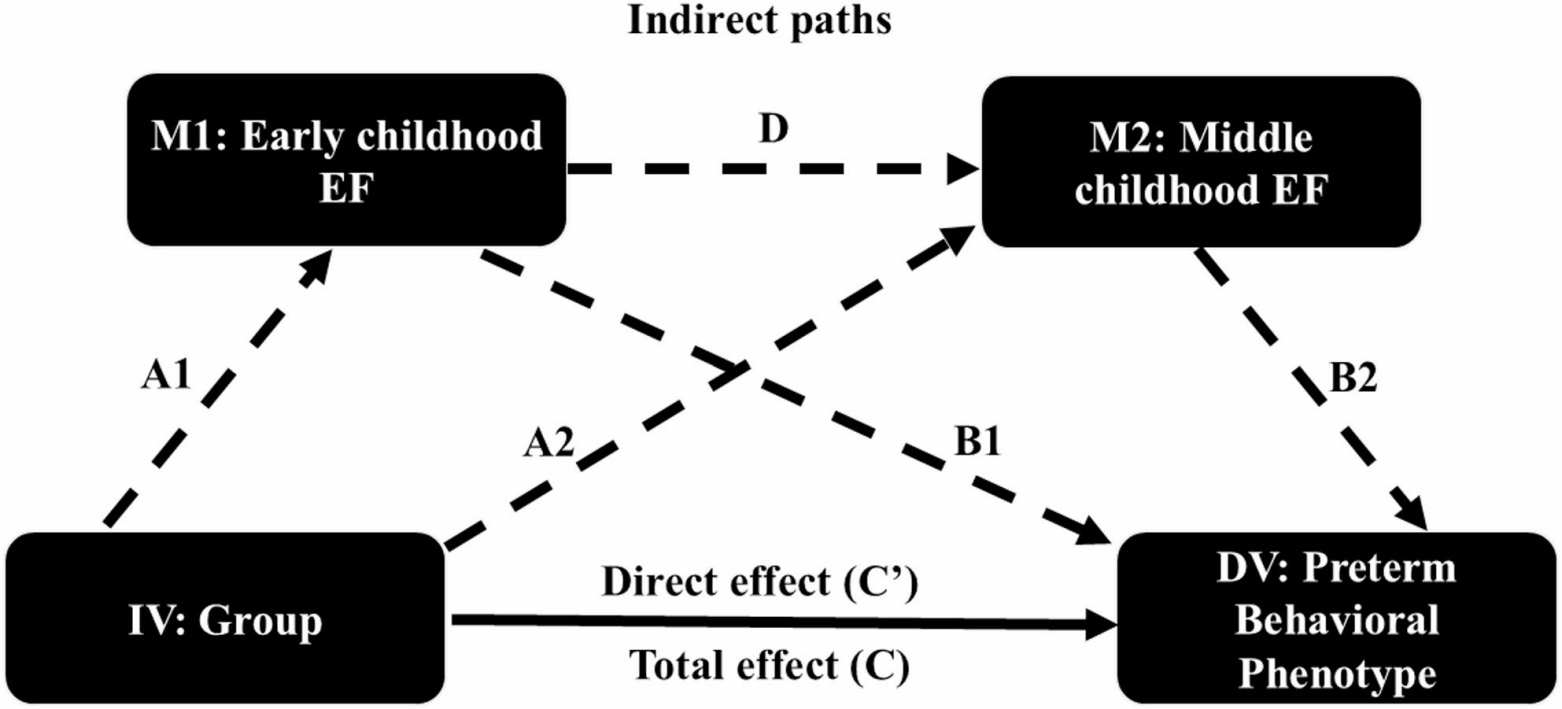
Serial mediation: Associations between group and the Preterm Behavioral Phenotype via Executive Function (EF) Across Childhood. Paths A1 and A2 represent the association between study group (IV) and EF in early childhood (M1) and middle childhood (M2), respectively. Path D represents the association between EF in early childhood (M1) and EF in middle childhood (M2). Paths B1 and B2 represent the association between EF in early childhood (M1) and middle childhood (M2) with Preterm Behavioral Phenotype outcome (DV), respectively. Serial mediation analysis will simultaneously test three indirect pathways: indirect effect 1 via early childhood EF [A1 → B1]; indirect effect 2 via middle childhood EF [A2 → B2]; and indirect effect 3 via EF across childhood [A1 → D → B2]. IV, independent variable; DV, dependent variable; M1, mediator 1; M2, mediator 2

Data analysis was performed in SPSS (version 29). Continuously-distributed variables were screened for significant outliers (> 3 standard deviations) and non-normal distributions. One participant had a significant outlier on the Shape School shifting/flexibility condition. As between-groups differences in flexibility/shifting were identical when this value was winsorized, original data is reported. Partial data were estimated using multiple imputation with predictive mean matching. Data analyses were performed in three steps: First, EF and PBP outcomes were compared between FT, VPT, and WMI children using linear mixed-effects models (LMMs) with post-hoc comparisons that were Bonferroni-corrected for multiple comparisons. LMMs were first performed with group as a fixed effect and family clustering as a random effect with random intercept to account for nested data among VPT twins and triplets. When the random covariance parameter was redundant (i.e., the parameter was zero and could not be estimated), models were run with fixed effects only which did not change the pattern of results. Between-groups differences in EF outcomes were then examined after covariate adjustment for sex and the social disadvantage composite. PBP outcomes were adjusted for sex and social disadvantage, and additionally the maternal psychosocial distress index to account for the heritability of mood/affective symptoms. Given the higher rates of severe cognitive impairment in VPT and WMI children (see Table 1), sensitivity analysis was performed for EF outcomes excluding children with FSIQ < 70. Second, residualized-change LMMs were used to examine the stability of EF from early to middle childhood and to identify the key socioenvironmental and maternal psychosocial factors in early childhood [21] that related to change in EF over time. In these models, EF at age 9–10 years was regressed on EF at age 5 years to residualize the dependent variable [73]. Variability in EF outcome that is left unexplained by prior EF can be interpreted as the variability due to change and which can be explained by other factors [74, 75]. Third and last, serial mediation analysis was conducted using the PROCESS 4.0 macro to investigate the extent that EF during childhood explains the association between study group and PBP outcomes at age 9–10 years (Fig. 1). Group membership was defined as the independent variable and entered as a multi-categorical variable with dummy coding to generate estimates for both the VPT group and the WMI group relative to the FT group in the same model. EF at age 5 years was defined as the first mediator and EF at age 9–10 years was defined as the subsequent mediator in the indirect pathway. PBP outcomes were used as the dependent variable. As recommended [76], significance of the indirect effects was determined using 95% confidence intervals with bootstrapping (5000 samples with replacement). Mediation models were performed with robust standard errors and with covariate adjustment for the social disadvantage composite and maternal psychosocial distress index at the 9–10 year follow-up. Supplementary mediation models were also performed with infant socioemotional problems at age 2 years included as covariate factors on the basis that infant socioemotional problems were correlated with mental health outcomes in later childhood (see Table S2, Supplementary Material, for ITSEA correlations with key PBP outcomes of interest).

## Results

### Executive function outcomes

#### Early childhood EF

Between-group differences were found for global EF (*p*=.02) and inhibitory control (*p*=.04) at age 5 years, with VPT and WMI children performing less well than FT children (Table 2). Findings for global EF persisted after accounting for sex and social disadvantage (*p*=.04). Additionally, birth-group differences in global EF (*p*=.03) and inhibitory control (*p*=.03) remained significant after excluding children with severe cognitive impairment at age 5 years. In contrast, FT, VPT, and WMI children had similar short-term memory and shifting/flexibility skills at age 5 years (*p*>.05).

**Table 2:** Executive function (EF) outcomes for full-term (FT), very preterm (VPT), and high-grade white matter injury (WMI) children (*n* = 202)

M (SE)	FT (*n* = 79)	VPT (*n* = 79)	WMI (*n* = 44)	F	Marginal *R*^2^	*p*	*p* ^a^	*p* ^b^
EF at Age 5–6 Years								
Global EF	0.16 (0.12)	-0.17 (0.11)	-0.26 (0.15)	4.71	0.05	0.02	0.04	0.03
Short-term memory	46.86 (1.42)	43.99 (1.32)	42.89 (1.59)	3.20	0.03	0.08	0.14	0.18
Inhibitory control	0.92 (0.06)^c^	0.76 (0.05)^c^	0.79 (0.06)	4.69	0.05	0.04	0.05	0.03
Shifting/flexibility	0.24 (0.05)	0.22 (0.04)	0.17 (0.07)	1.36	0.02	0.38	0.45	0.25
EF at Age 9–10 Years								
Global EF	0.36 (0.10)^c, d^	-0.07 (0.11)^c, e^	-0.61 (0.15)^d, e^	15.96	0.14	< 0.001	< 0.001	< 0.001
Working memory	95.78 (2.02)^d^	89.24 (2.37)	82.71 (2.92)^d^	8.15	0.08	0.001	0.001	0.001
Inhibitory control	87.97 (1.27)^c, d^	83.38 (1.34)^c, e^	76.22 (1.82)^d, e^	15.46	0.14	< 0.001	< 0.001	< 0.001
Shifting/flexibility	90.59 (1.34)^d^	86.54 (1.35)	82.32 (1.86)^d^	7.07	0.07	0.001	0.001	0.02

All pair-wise comparisons corrected for multiple comparisons using embedded Bonferroni correction. Marginal R^2^ provided as a measure of effect size for the fixed effect of group

^a^ Adjusted for sex and social disadvantage composite

^b^ Excluding children with severe cognitive impairment (FSIQ < 70)

^c^ FT vs. VPT *p*<.05

^d^ FT vs. WMI *p*<.05

^e^ VPT vs. WMI *p*<.05

#### Middle childhood EF

There were clear between-groups differences across all EF domains at age 9–10 years (all *p*≤.001, Table 2). The stronger association between group and EF abilities at age 9–10 appeared to be driven by WMI children obtaining significantly lower global EF and inhibitory control scores than both VPT children and FT children (Bonferroni-corrected post-hoc *p*<.05). Birth-group differences persisted for all EF domains after covariate adjustment for sex and social disadvantage (all *p*≤.001) and when children with severe cognitive impairment at age 9–10 years were excluded from the analysis (all *p*≤.02).

#### Early childhood factors associated with EF in middle childhood

Bivariate correlations between early childhood socioenvironmental factors and subsequent EF outcomes are shown in Table S3 (Supplemental Material). Briefly, social disadvantage, maternal depression symptoms, stressful/traumatic life events, and maternal IQ assessed at the 5-year follow-up were correlated with global EF outcomes at age 9–10 years (*r* range − 0.39–0.28, all *p*<.05). These factors were carried forward for inclusion as independent variables in multivariable residualized change LMMs.

As shown in Table 3, global EF at age 5 years was positively associated with subsequent global EF at age 9–10 years (*p*=.04) after adjustment for key covariate factors. Among the key covariate factors that were correlated with EF outcome, exposure to greater social disadvantage at age 5 years was independently related to lower EF abilities at age 9–10 years (*p*=.002) whereas associations with maternal depression symptoms and stressful/traumatic life events at age 5 years were no longer significant (*p*>.05). After accounting for baseline EF at age 5 years and covariate factors, VPT birth (*p*=.01) and WMI (*p*<.001) remained significant risk factors for lower EF abilities by age 9–10 years. There were no interactions between group and social disadvantage at age 5 years (*p*=.31), group and baseline EF at age 5 years (*p*=.51), or between social disadvantage and baseline EF at age 5 years (*p*=.50).

**Table 3:** Summary of linear-mixed effects model relating group, global executive function at age 5 years, and maternal and family factors at the 5-year follow-up to subsequent global executive function outcome at age 9–10 years (*n* = 202)

Independent variables	Estimate	SE	t	*p*	95% Confidence interval	
					Lower	Upper
Group^a^						
* WMI*	-0.81	0.17	-4.83	< 0.001	-1.13	-0.48
* VPT*	-0.34	0.14	-2.46	0.01	-0.61	-0.07
Global EF at age 5 years	0.23	0.11	2.10	0.04	0.01	0.45
Social disadvantage composite at age 5 years	-0.29	0.09	-3.23	0.002	-0.47	-0.11
Maternal depression symptoms at age 5 years	-0.02	0.01	-1.62	0.11	-0.03	0.003
Stressful/traumatic life events at age 5 years	0.001	0.01	-0.11	0.92	-0.02	0.01

^a^ Intercepts for WMI and VPT groups are relative to the FT group

Of note, maternal FSIQ was highly correlated with the social disadvantage composite (*r* = − .62, *p*<.001). Nevertheless, for completeness, a supplementary residualized-change LMM including maternal FSIQ is provided in Table S4 (Supplementary Material). These results show that maternal FSIQ was not significant (*p*=.39) over and above social disadvantage (*p*=.04), VPT birth (*p* = 01), or WMI (*p* < 001). Residualized-change LMMs for short-term/working memory, inhibitory control, and shifting/flexibility outcomes at age 9–10 years are shown in Tables S5-S7 (Supplementary Material), with similar conclusions regarding associations with birth group and social disadvantage. Among VPT and WMI children, infant clinical factors including gestational age, birthweight, and infant medical risk index were not significant (all *p*>.05) in multivariable models that included social disadvantage and maternal psychosocial distress at age 9–10 years as covariate factors (Tables S8-9).

### Preterm Behavioral Phenotype (PBP) outcomes

Table 4 shows the PBP outcomes between WMI, VPT, and FT children age 9–10 years. Regarding parent-informant ratings, there was a significant between-groups difference in ADHD-inattentive symptoms (*p*=.006, covariate adjusted *p*=.002), with WMI children obtaining higher ratings than FT children (Bonferroni-corrected post-hoc *p*<.05). WMI children also obtained higher ratings for social communication-interaction challenges, but only after covariate adjustment (*p*=.01). In terms of child-informant measures, between-groups differences were observed in a step-wise manner for the PBP composite and social perception tasks measuring affect recognition and theory of mind (all *p*<.001). Specifically, WMI children showed poorer outcomes in these areas compared to both VPT and FT children, with additional differences observed between VPT and FT children (all Bonferroni-corrected post-hoc *p*<.05). WMI children also self-reported more ADHD-inattentive symptoms than FT children (*p* = .03, covariate adjusted *p*=.02). There were no between-groups differences in any of the other PBP outcomes (all *p*>.05).

**Table 4:** Preterm Behavioral Phenotype (PBP) outcomes at age 9–10 years for full-term (FT), very preterm (VPT), and high-grade white matter injury (WMI) children (*n* = 202)

PBP at 9-10 years, M (SE)	FT (*n* = 79)	VPT (*n* = 79)	WMI (*n* = 44)	F	Marginal *R*^2^	*p*	*p* ^a^
Parent informant							
PBP composite score	-0.18 (0.20)	0.09 (0.21)	0.27 (0.27)	0.98	0.01	0.38	0.09
Internalizing symptoms	48.63 (1.20)	48.55 (1.30)	49.89 (1.63)	0.25	< 0.01	0.78	0.63
Anxiety symptoms	13.36 (1.23)	13.00 (1.33)	10.67 (1.67)	1.04	0.01	0.37	0.44
Social communication-interaction differences	52.75 (1.31)	55.80 (1.43)	56.56 (1.78)	1.95	0.02	0.15	0.01
ADHD-Hyperactivity symptoms^b^	53.26 (1.58)	54.27 (1.64)	56.58 (2.15)	0.82	0.01	0.45	0.21
ADHD-Inattention symptoms	54.84 (1.78)^d^	59.95 (1.91)	64.27 (2.42)^d^	5.28	0.05	0.006	0.002
Child informant							
PBP composite score^b^	-0.56 (0.15)^c, d^	0.14 (0.16)^c, e^	0.77 (0.21)^d, e^	14.15	0.13	< 0.001	< 0.001
Internalizing symptoms	57.38 (0.82)	59.89 (0.84)	59.13 (1.11)	2.44	0.03	0.09	0.06
Social perception – affect recognition	54.48 (1.16)^c, d^	50.10 (1.27)^c, e^	44.75 (1.58)^d, e^	12.61	0.11	< 0.001	< 0.001
Social perception – theory of mind	23.11 (0.53)^c, d^	21.09 (0.53)^c, e^	18.17 (0.71)^d, e^	15.95	0.14	< 0.001	< 0.001
ADHD-Inattention symptoms	58.87 (0.80)^d^	60.70 (0.83)	62.39 (1.08)^d^	3.71	0.04	0.03	0.02

All pair-wise comparisons corrected for multiple comparisons using embedded Bonferroni correction. Marginal R^2^ provided as a measure of effect size for the fixed effect of group

^a^ Adjusted for sex, social disadvantage composite, and maternal psychosocial distress index. Findings for social perception tasks were also significant (*p*<.001) when excluding children with IQ < 70 at age 9–10 years

^b^ Lower scores on social perception tasks indicate greater problems. Higer scores on all other measures indicate greater problems. Social perception tasks were reverse-scored prior to computing the child-informant PBP PCA score

^c^ FT vs. VPT *p*<.05

^d^ FT vs. WMI *p*<.05

^e^ VPT vs. WMI *p*<.05

### Associations between EF and the PBP

Bivariate associations between EF measures and the PBP are shown in Table 5. Of particular interest, poorer global EF and short-term or working memory skills at ages 5 and 9–10 years were correlated with parent-informant social communication-interaction differences, child-informant PBP composite ratings, and theory of mind skills at age 9–10 years (all *p*<.05). These domains were carried forward for serial mediation analysis per the following criteria: (a) between-groups differences observed in EF and PBP domains (Tables 2 and 4, respectively), (b) significant correlations between EF measures within a domain over time (Table S1), and (c) significant correlations between EF and PBP domains (Table 5).

**Table 5:** Bivariate correlations between executive function (EF) and Preterm Behavioral Phenotype (PBP) domains (*n* = 202)

PBP at age 9–10 Years	EF at age 5 Years				EF at age 9–10 years			
	Global EF	Short-term memory	Inhibitory control	Shifting/flexibility	Global EF	Working memory	Inhibitory control	Shifting/flexibility
Parent-informant								
PBP composite score	-0.14	-0.13	-0.10	-0.05	-0.30***	-0.23**	-0.23**	-0.27***
Internalizing symptoms	0.03	0.02	0.01	0.04	-0.16*	-0.10	-0.15*	-0.14*
Anxiety symptoms	-0.09	-0.04	-0.06	-0.07	-0.17*	-0.09	-0.13	-0.18*
Social communication-interaction differences	-0.25**^a^	-0.20*^a^	-0.16	-0.13	-0.39***^a^	-0.29**^a^	-0.30***	-0.35***
ADHD-Hyperactivity symptoms	-0.08	-0.13	-0.05	0.02	-0.19*	-0.18*	-0.14*	-0.14
ADHD-Inattention symptoms	-0.17	-0.14	-0.12	-0.08	-0.28***	-0.25***	-0.16*	-0.24***
Child-informant								
PBP composite score	-0.23**^a^	-0.21*^a^	-0.10	-0.16	-0.45***^a^	-0.36***^a^	-0.33***	-0.38***
Internalizing symptoms	-0.10	-0.08	-0.03	-0.08	-0.23**	-0.21*	-0.13	-0.21**
Social perception – affect recognition	0.17	0.18*	0.07	0.10	0.32***	0.22*	0.30***	0.24***
Social perception – theory of mind	0.28**^a^	0.25*^a^	0.15	0.16	0.41***^a^	0.33**^a^	0.29***	0.37***
ADHD-Inattention symptoms	-0.11	-0.09	-0.02	-0.1	-0.30***	-0.26**	-0.21**	-0.25***

^*a*^ Domains that satisfy criteria for inclusion in serial mediation analysis

* p < .05, ** *p* < .01, *** *p* < .001

#### Parent-informant social communication-interaction differences

**Fig. 2:**
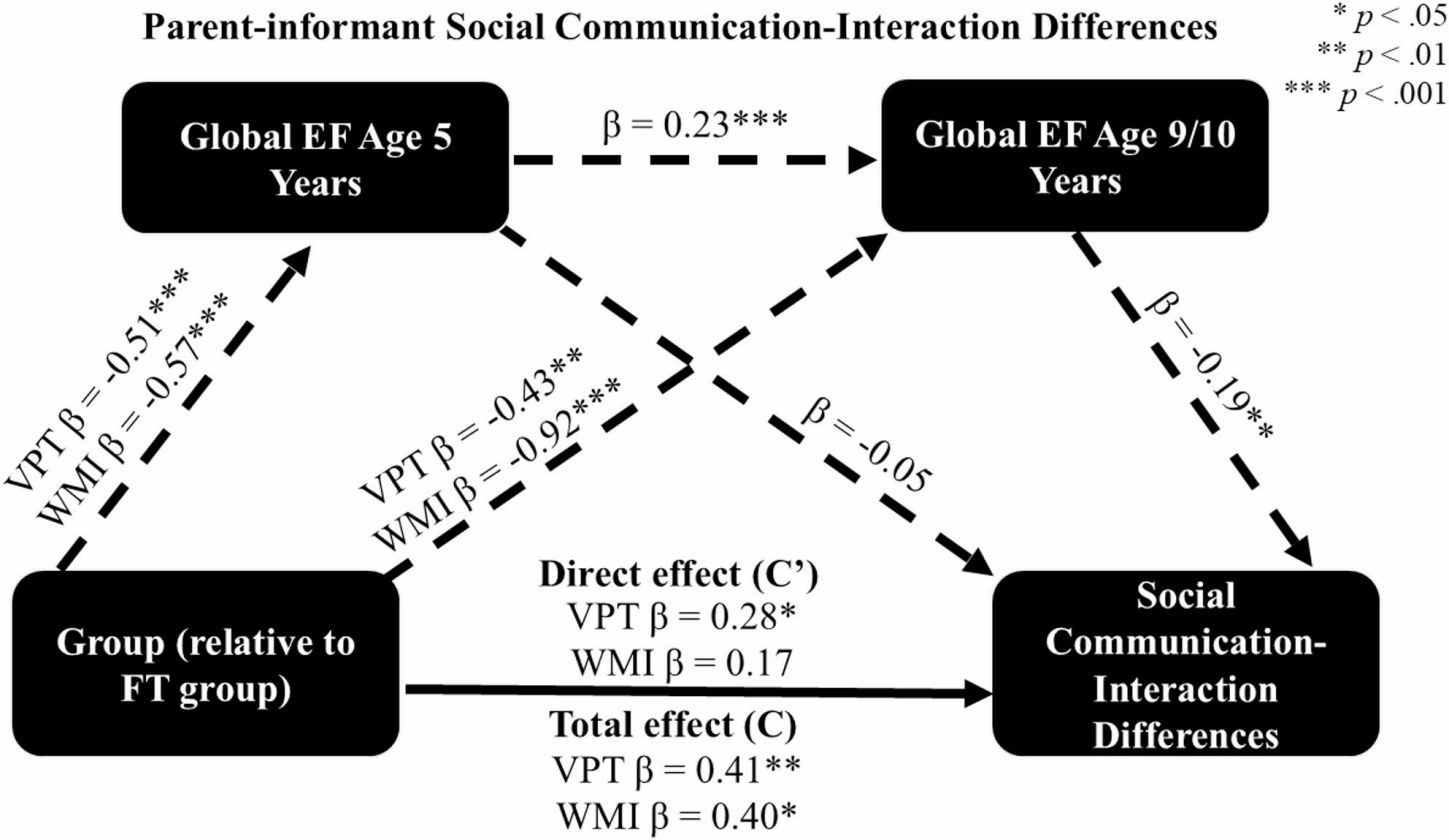
Serial Mediation Analysis: Path estimates linking group and executive function (EF) with social communication-interaction differences. Social communication-interaction differences at age 9–10 years were partially or completely mediated by via two indirect pathways linking group with global EF from age 5 years to age 9–10 years and with EF at age 9–10 years. Model is adjusted for the covariate factors social disadvantage and maternal psychosocial stress at the 9–10 year follow-up

As shown in Table 6; Fig. 2, the association between VPT birth and social communication-interaction differences was partially mediated (direct effect 95% CIs: 0.01–0.55) by two indirect pathways involving poorer EF from age 5 to 9–10 years (95% CIs: 0.004–0.05) and EF at age 9–10 years (95% CIs: 0.02–0.16) (total proportion mediated 24%). A similar and stronger pattern of results was found for the WMI group, such that these two indirect pathways completely mediated association between WMI and social communication-interaction problem outcomes (direct effect 95% CIs: -0.20–0.54) with twice the amount of variance explained (total proportion mediated 48%) than in the VPT group. For both groups, the pathway involving poorer EF at age 9–10 years accounted for a larger proportion of the mediation (20–43%) than the pathway involving poorer EF from age 5 to 9–10 years (5%).

**Table 6:** Summary of serial mediation analysis testing global executive function (EF) as a mediator between group and Preterm Behavioral Phenotype outcomes (*n* = 202)

Effects:	Total effect	Direct effect	Indirect effect 1: EF at age 5 years	Indirect effect 2: EF at age 9–10 years	Indirect effect 3: EF from age 5 to 9–10 years		Conclusion
	IV → DV	IV → DV controlling for M1 and M2	IV → M1→ DV	IV → M2→ DV	IV → M1→ M2 → DV		
*Model: Parent-informant social communication-interaction differences mediated by global EF*							
VPT	0.41 ± 0.13 (0.15–0.66)	0.28 ± 0.14 (0.01–0.55)	0.03 ± 0.03 (-0.02–0.11)	0.08 ± 0.04 (0.02–0.16)^a^	0.02 ± 0.01 (0.004–0.05)^a^		Partial mediation
*Proportion mediated (%)*				*19.5*	*4.9*		
WMI	0.40 ± 0.17 (0.07–0.73)	0.17 ± 0.19 (-0.20–0.54)	0.03 ± 0.04 (-0.03–0.11)	0.17 ± 0.06 (0.06–0.30)^a^	0.02 ± 0.01 (0.006–0.05)^a^		Full mediation
*Proportion mediated (%)*				*42.5*	*5.0*		
*Model: Child-informant PBP composite score mediated by global EF*							
VPT	0.56 ± 0.13 (0.31–0.82)	0.41 ± 0.14 (0.14–0.67)	0.01 ± 0.03 (-0.07–0.07)	0.11 ± 0.05 (0.04–0.21)^a^	0.03 ± 0.02 (0.01–0.07)^a^		Partial mediation
*Proportion mediated (%)*				*19.6*	*5.4*		
WMI	0.99 ± 0.20 (0.60–1.37)	0.69 ± 0.20 (0.29–1.09)	0.02 ± 0.04 (-0.06–0.09)	0.24 ± 0.08 (0.11–0.41)^a^	0.04 ± 0.02 (0.01–0.07)^a^		Partial mediation
*Proportion mediated (%)*				*24.2*	*4.0*		
*Model: Child-informant theory of mind mediated by global EF*							
VPT	-0.50 ± 0.12 (-0.72 – -0.27)	-0.33 ± 0.12 (-0.57 – -0.10)	-0.06 ± 0.03 (-0.13 – -0.01)^a^	-0.08 ± 0.04 (-0.16 – -0.02)^a^	-0.02 ± 0.01 (-0.05 – -0.01)^a^		Partial mediation
*Proportion mediated (%)*			*12.0*	*16.0*	*4.0*		
WMI	-1.05 ± 0.23 (-1.50 – -0.60)	-0.78 ± 0.23 (-1.23 – -0.33)	-0.07 ± 0.03 (-0.13 – -0.01)^a^	-0.17 ± 0.06 (-0.31 – -0.06)^a^	-0.03 ± 0.01 (-0.05 – -0.01)^a^		Partial mediation
*Proportion mediated (%)*			*6.7*	*16.2*	*2.9*		

IV, independent variable; DV, dependent variable; M1, mediator 1; M2, mediator 2. Standardized estimates ± standard error and 95% confidence intervals shown. Bootstrapping (5000 samples) used to construct 95% confidence intervals for the indirect effects. Group (FT, VPT, WMI) entered as a multi-categorical independent variable using dummy coding; estimates for WMI and VPT groups are relative to the FT group. Proportion mediated (%) is calculated as indirect effect/total effect x 100. All models adjusted for social disadvantage composite and maternal distress index at the 9–10 year follow-up

^a^ Indirect effects remained significant when additionally adjusted for infant ITSEA dysregulation and social competency problems at age 2 years (see Table S10, Supplementary Material where results are reported in full)

#### Child-informant PBP composite scores

EF served as a partial mediator of child-informant PBP composite scores for both VPT and WMI children via two indirect pathways (Table 6, Figure S1). In the VPT group, the two indirect pathways involving poorer EF from age 5 to 9–10 years (95% CIs: 0.01–0.07) and EF at age 9–10 years (95% CIs: 0.04–0.21) partially mediated the association between VPT birth and higher overall PBP composite scores (direct effect 95% CIs: 0.14–0.67) (total proportion mediated 25%). A similar pattern of results was found in the WMI group (total proportion mediated 28%, Table 6 and Figure S1). For both groups, the pathway involving poorer EF at age 9–10 years accounted for a larger proportion of the mediation (20–24%) than the pathway involving poorer EF from age 5 to 9–10 years (4–5%).

#### Child-informant theory of mind skills

EF served as a partial mediator of theory of mind ability for both VPT and WMI children via all three indirect pathways (Table 6, Figure S2). In the VPT group, the three indirect pathways involving EF at age 5 years (95% CIs: -0.13 – -0.01), EF from age 5 to 9–10 years (95% CIs: -0.05 – -0.01), as well as EF at age 9–10 years (95% CIs: -0.16 – -0.02) partially mediated the association between VPT birth and lower theory of mind abilities (direct effect 95% CIs: -0.57 – -0.10) (total proportion mediated 32%). A similar but slightly weaker pattern of results was found in the WMI group (total proportion mediated 26%, Table 6 and Figure S2). EF at age 9–10 years overall accounted for a larger proportion of the mediating effect in both groups (16%) than the other indirect pathways (EF at age 5 years: 7–12%; EF from age 5 to 9–10 years: 3–4%).

#### Supplementary serial mediation analyses

As shown in Table S10 (Supplementary Material), the mediating role of EF during childhood on PBP problems remained significant when models also included ITSEA dysregulation and social competency ratings at age 2 years as covariate factors. Supplementary analyses were also undertaken to examine the mediating role of short-term/working memory as a specific component of EF, with similar results (Table S11). Briefly, short-term/working memory development partially mediated the effect of group on child-informant PBP and theory of mind outcomes via indirect pathways involving short-term/working memory ability from age 5 to 9–10 years and at age 9–10 years. In contrast, short-term/working memory did not serve as a mediator for parent-informant social communication-interaction differences.

## Discussion

The current study described the EF outcomes of WMI, VPT, and FT children at ages 5 and 9–10 years, and examined the extent that EF development during childhood served as a neurocognitive mechanism for the PBP. In line with prior research [45, 77], we found that VPT and WMI children showed challenges in global EF, as well as domain-specific challenges in inhibitory control, compared to FT children in early childhood. In contrast, short-term memory and shifting/flexibility outcomes were similar across the study groups. While we have previously reported differences in short-term memory and shifting/flexibility between VPT and FT children using the same EF battery at age 5 years [21], the current analysis included a larger group of FT children with higher rates of family socio-demographic and neighborhood disadvantage than the FT group included in our previous work. Other research has shown that social disadvantage is more strongly related to delays in early-developing memory skills, as well as more complex EFs that involve the flexible adjustment to changing task demands, than other aspects of EF [78–80]. FT children in the current study sample may, therefore, have shown lower short-term memory and shifting/flexibility abilities at age 5 years than might be expected, reducing the magnitude of the between-groups difference in early childhood for these EF domains. However, it is also important to note that VPT birth has not been consistently associated with shifting/flexibility outcomes, with discrepant findings across studies attributed to differences in the neuropsychological properties of the EF task used, age at assessment, and degree of prematurity [18, 77, 81]. Nonetheless, by age 9–10 years, between-groups differences were observed across all EF domains, with WMI children showing the most impaired EF outcomes. Our findings support the deficit model of EF development [24], such that VPT and WMI children demonstrate persistent global EF difficulties with no evidence of catch-up to FT children at least into middle childhood. Prior research has similarly reported difficulties across multiple EF skills in VPT children [16–18, 20], including in VPT children with diffuse moderate/severe neonatal white matter abnormalities assessed using qualitative ratings [82]. Our results build upon Woodward et al. [82] to further suggest that EF challenges in WMI children become more pronounced over time relative to VPT children and FT children. As such, VPT children and WMI children, in particular, are in need of early and ongoing cognitive interventions that include strategies to support EF development [83, 84].

Our work has previously shown that exposure to social disadvantage, maternal mood/affective symptoms, and less supportive parenting behavior is related to executive dysfunction in VPT children at age 5 years [21]. We extend our prior findings by examining whether early social and family risk factors also relate to individual differences in EF development by age 9–10 years using residualized change models that controlled for prior EF in early childhood. We found that a similar set of early childhood risk factors including social disadvantage, maternal depression symptoms, and stressful/traumatic life events correlated with lower EF ability by age 9–10 years. In residualized change models, exposure to greater social disadvantage at age 5 years was related to declining EF ability by age 9–10 years after accounting for prior EF, whereas associations concerning maternal depression and stressful/traumatic life events were attenuated. Consistent with our results, social disadvantage is a well-established risk factor for executive dysfunction across development [79]. While maternal depression has also been associated with reduced growth in EF development [85], our finding regarding the stronger association of social disadvantage on EF development potentially reflects the fact that we examined cumulative exposure to multiple social disadvantage factors in a socially diverse sample, making the signal of social disadvantage more detectable and robust. We also note that the results of residualized change models are consistent with the dual hazard hypothesis [86], positing that biological and social risk factors associated with prematurity contribute to adverse neurodevelopmental outcomes. Specifically, after accounting for VPT birth and prior EF ability, high-grade perinatal WMI and early childhood exposure to social disadvantage independently posed additional risk for EF challenges over time. These findings highlight that both neurological and social factors increase the likelihood of executive dysfunction in preterm populations. Larger samples are, however, needed to understand whether perinatal neurological risk differentially increases susceptibility to exposure to socioenvironmental adversity in the pathway towards executive dysfunction.

In addition to EF challenges, VPT and WMI children had higher levels of parent- and child-identified social communication-interaction differences and ADHD-inattentive symptoms at age 9–10 years compared to FT children. WMI children obtained the most impaired ratings across these domains. This pattern of results is highly consistent with the well-established link between VPT birth and ADHD and Autism in childhood [30, 42, 87], and further suggests that WMI children are at heightened risk for the PBP. Among the PBP domains, prominent challenges were observed on direct measures of affect recognition and theory of mind in VPT and WMI children. Multiple studies have found that VPT children exhibit difficulties in social cognition which, in turn, impact social interaction capabilities and social adjustment outcomes [88–90]. In contrast to the extant literature [30, 91–93], our study did not find evidence of between-group differences on parent- or child-informant measures of internalizing/anxiety symptoms at age 9–10 years. There is some suggestion that internalizing symptoms may be more prominent in children born at earlier gestational ages and/or that internalizing symptoms become more impairing in adolescence [94]. Ongoing neurodevelopmental follow-up into adolescence will, therefore, be important to determine whether between-groups differences in internalizing symptoms emerge later in the risk trajectory for the PBP.

The final objective of this study was to examine the extent that executive dysfunction during childhood placed VPT and WMI children at increased risk for the PBP at age 9–10 years. Serial mediation analysis showed that executive dysfunction partially accounted for PBP problems across parent-informant social communication-interaction and child-informant PBP composite and theory of mind domains, in VPT and WMI children relative to FT children. We also found that executive dysfunction fully accounted for the higher levels of parent-informant social communication-interaction differences in WMI children, suggesting stronger links between executive dysfunction and social communication-interaction differences in the context of WMI. Associations between executive dysfunction and parent-report ratings of social responsiveness problems have been found in EPT children at age 10 years [42]. These collective findings are consistent with frameworks suggesting that executive dysfunction underpins the core features of social communication-interaction challenges, including rigid social behaviors and difficulty adapting to dynamic changes in the social environment [34, 95]. Supplementary analysis further revealed that short-term/working memory development was important for the child-informant PBP and, specifically, theory of mind ability. Working memory, and it’s precursor short-term memory, is one of the earliest developing components of EF [78] and is regarded as a critical aspect of cognition necessary for holding and integrating different perspectives in mind during social interactions [96, 97].

Serial mediation findings were overall consistent in terms of two significant indirect pathways involving poorer EF development from age 5 to 9–10 years, and lower EF ability at age 9–10 years, in relation to the PBP. For all models, EF challenges at age 9–10 years accounted for the largest proportion of the mediation, likely reflecting the concurrent assessment of EF and the PBP at the 9–10 year follow-up. Cross-sectional associations between executive dysfunction and socioemotional problems have been found in studies of preterm children [41–45]. Importantly, even after accounting for the cross-sectional association between EF and the PBP at age 9–10 years, the indirect pathway involving EF development from age 5 to 9–10 years was still significant. In addition, the indirect pathway involving early EF at age 5 years was also significant for theory of mind outcomes, with this pathway explaining a somewhat similar proportion of variance (12%) as did EF at age 9–10 years (16%) in VPT children. We also note that findings persisted when infant socioemotional problems that preceded the assessment of EF and the PBP were accounted for. Thus, while EF demonstrates concurrent associations with the PBP in middle childhood, early and persisting challenges in EF also appear to play a role in the evolution of the PBP. These findings indicate that early intervention training paradigms targeting EF development could be important to improve the mental health outcomes of VPT and WMI children.

The current study has numerous strengths including a prospective longitudinal study design, a larger group of WMI children compared to prior studies [25, 26, 28], and parent- and child-informant assessment of the PBP. Study findings should also be interpreted in light of the limitations. First, different EF tasks were used at the 5 and 9–10 year assessments, potentially adding method variance and under-estimating longitudinal associations in EF. The use of different EF tasks also precluded formal multilevel analysis which requires repeated measurements using the same scale at each timepoint [98]. However, the EF tasks used at each timepoint were developmentally appropriate and generally tapped similar constructs. Second, the study did not have direct assessment of maternal EF. Although we included a measure of maternal intellectual ability, and maternal intellectual ability and EF are correlated [99], maternal EF is more directly related to child EF [99]. We may, therefore, have underestimated heritable influences on child EF. Third, while our sample size is larger than other prospective studies of EF outcomes in VPT children and adolescents [23, 26, 28], sample size was modest. We also acknowledge that the 9–10 year assessments of EF and the PBP were concurrent, making it difficult to untangle bi-directional associations at this timepoint. We note that previous studies in VPT children and adolescents have conducted single mediation analysis using concurrent EF and socio-emotional assessments [45, 46], whereas our study conducted serial mediation analysis that included two prospective assessments of EF starting in early childhood. In supplementary analyses, we also controlled for infant socioemotional problems that preceded the assessment of EF in early childhood which other studies have not done [45, 46]. Future research may benefit from assessing EF over time using more recent standardized cognitive toolboxes that include lifetime EF tasks suitable for use starting in early childhood in larger samples of VPT and WMI children with assessments continuing into adolescence.

## Conclusions

VPT and WMI children had poorer EF outcomes than FT children in early and middle childhood, suggesting persistent EF challenges that do not catch up to FT children at least during this stage of development. Furthermore, WMI children showed the lowest EF ability that became more pronounced over time relative to VPT and FT children. At the 9–10 year follow-up, VPT and WMI children also had higher levels of PBP problems, with social communication-interaction differences and ADHD-inattentive symptoms observed across parent- and child-informants. Serial mediation analysis indicated that EF challenges during childhood placed VPT and WMI children at increased risk of the PBP via two indirect pathways involving altered EF development from age 5 to 9–10 years and lower EF abilities at age 9–10 years. Importantly, findings were robust such that results persisted after accounting for infant socioemotional problems and childhood exposure to social disadvantage and maternal psychosocial distress. These collective findings highlight that VPT and WMI children are important groups of children in need of early identification and referral to follow-up services that support cognitive development. While early developmental intervention programs implemented in preterm populations have typically focused on general cognitive development, strategies to include paradigms that more specifically target or train EFs may be important to improve EF outcomes and reduce psychopathology risk in later childhood.

## Supplementary Information

Supplementary Material 1: Table S1. Summary of tasks and questionnaires used to assess key domains of interest. Table S2. Bivariate correlations between Infant and Toddler Social and Emotional Assessment (ITSEA) problems at age 2 years and Preterm Behavioral Phenotype (PBP) outcomes with significant between-groups differences at age 9–10 years (*n* = 202). Table S3. Bivariate correlations between socioenvironmental factors in early childhood and executive function (EF) outcomes at ages 5 and 9–10 years (*n* = 202). Table S4. Summary of linear-mixed effects model additionally including maternal IQ as a predictor of global executive function outcome at age 9–10 years (*n* = 202). Table S5. Summary of linear-mixed effects model relating group, short-term memory at age 5 years, and maternal and family factors at the 5-year follow-up to working memory outcome at age 9–10 years (*n* = 202). Table S6. Summary of linear-mixed effects model relating group, inhibitory control at age 5 years, and maternal and family factors at the 5-year follow-up to inhibitory control outcome at age 9–10 years (*n* = 202). Table S7. Summary of linear-mixed effects model relating group, shifting/flexibility at age 5 years, and maternal and family factors at the 5-year follow-up to shifting/flexibility outcome at age 9–10 years (*n* = 202). Table S8. Bivariate correlations between infant clinical factors and primary outcome measures among the VPT and WMI groups. Table S9. Associations between infant clinical factors and primary outcome measures among the VPT and WMI groups after accounting for group and covariate factors. Table S10. Summary of serial mediation analysis testing global executive function (EF) as a mediator between group and Preterm Behavioral Phenotype (PBP) outcomes with additional adjustment for infant socio-emotional problems at age 2 years (*n* = 202). Table S11. Summary of serial mediation analysis testing short-term/working memory (WM) as a mediator between group and Preterm Behavioral Phenotype (PBP) outcomes (*n* = 202). Figure S1. Serial Mediation Analysis: Path estimates linking group and executive function (EF) with the Preterm Behavioral Phenotype. Results show that Preterm Behavioral Phenotype outcomes were partially mediated by via two indirect pathways linking group with global EF from age 5 years to age 9–10 years and with EF at age 9–10 years. Model is adjusted for social disadvantage and maternal psychosocial stress at the 9–10 year follow-up. Figure S2. Serial Mediation Analysis: Path estimates linking group and executive function (EF) with theory of mind skills. Results show that theory of mind outcomes were partially mediated by via three indirect pathways linking group with global EF at age 5 years, from age 5 years to age 9–10 years, and with EF at age 9–10 years. Model is adjusted for social disadvantage and maternal psychosocial stress at the 9–10 year follow-up.

## Data Availability

Data can be made available to qualified researchers by written request to Drs. C.D. Smyser and C.E. Rogers under the guidance of a formal data sharing agreement. Per NIH funding requirements, data related to the larger study will be shared on the open access NIMH Data Archive repository and made publicly available at the conclusion of the larger study.

## Abbreviations

ADHD: Attention Deficit/Hyperactivity Disorder
cPVL: Cystic periventricular leukomalacia
EF: Executive function
FT: Full term
FSIQ: Full scale intelligence quotient
GA: Gestational age
ITSEA: Infant and Toddler Social and Emotional Assessment
IVH: Intraventricular hemorrhage
LMM(s): Linear mixed-effects model(s)
MRI: Magnetic resonance imaging
NICU: Neonatal intensive care unit
PBP: Preterm Behavioral Phenotype
PSI: Parent Stress Index
PCA: Principal Component Analysis
PHH: Post-hemorrhagic hydrocephalus
VPT: Very preterm
WAIS-III: Weschler Adult Intelligence Scale-3rd Edition
WASI-II: Weschler Abbreviated Scale of Intelligence-2nd Edition
WMI: White matter injury
WPPSI-III: Weschler Preschool and Primary Scale of Intelligence-3rd Edition
WTAR: Weschler Test of Adult Reading

## References

[1] Roush K. March of Dimes Gives United States a D + for Preterm Birth Rates. AJN Am J Nurs. 2025;125(3):14. 10.1097/01.NAJ.0001108276.35474.e1.41261477

[2] Santhakumaran S, Statnikov Y, Gray D, Battersby C, Ashby D, Modi N, et al. Survival of very preterm infants admitted to neonatal care in England 2008–2014: time trends and regional variation. Arch Dis Child Fetal Neonatal Ed. 2017;103(3):208–15. 10.1136/archdischild-2017-312748 . PubMed PMID: 28883097. 10.1136/archdischild-2017-312748 PMC5916099 28883097

[3] Cheong JLY, Spittle AJ, Burnett AC, Anderson PJ, Doyle LW. Have outcomes following extremely preterm birth improved over time? Semin Fetal Neonatal Med. 2020;Long term outcomes following very preterm birth25(3):101114. 10.1016/j.siny.2020.101114 10.1016/j.siny.2020.101114 32451304

[4] Burnett AC, Anderson PJ, Lee KJ, Roberts G, Doyle LW, Cheong JLY, et al. Trends in Executive Functioning in Extremely Preterm Children Across 3 Birth Eras. Pediatrics. 2018;141(1):e20171958. 10.1542/peds.2017-1958. 29196505 10.1542/peds.2017-1958

[5] Baron IS, Rey-Casserly C. Extremely Preterm Birth Outcome: A Review of Four Decades of Cognitive Research. Neuropsychol Rev. 2010;20(4):430–52. 10.1007/s11065-010-9132-z. 20512418 10.1007/s11065-010-9132-z

[6] Behboodi S, Chaimani A, Benhammou V, Twilhaar ES, Johnson S, Zeitlin J, et al. Trends Over Time in Cognitive Outcomes of Children Born Very Preterm: A Systematic Review and Meta-Analysis. JAMA Pediatr. 2025;28. 10.1001/jamapediatrics.2025.2221. 10.1001/jamapediatrics.2025.2221 PMC12305443 40720136

[7] Pascal A, Govaert P, Oostra A, Naulaers G, Ortibus E, Van den Broeck C. Neurodevelopmental outcome in very preterm and very-low-birthweight infants born over the past decade: a meta-analytic review. Dev Med Child Neurol. 2018;60(4):342–55. 10.1111/dmcn.13675. 29350401 10.1111/dmcn.13675

[8] Van Naarden Braun K, Christensen D, Doernberg N, Schieve L, Rice C, Wiggins L, et al. Trends in the Prevalence of Autism Spectrum Disorder, Cerebral Palsy, Hearing Loss, Intellectual Disability, and Vision Impairment, Metropolitan Atlanta, 1991–2010. PLoS ONE. 2015;10(4):e0124120. 10.1371/journal.pone.0124120 . PubMed PMID: 25923140; PubMed Central PMCID: PMC4414511. 25923140 10.1371/journal.pone.0124120 PMC4414511

[9] Zablotsky B, Black LI, Maenner MJ, Schieve LA, Danielson ML, Bitsko RH, et al. Prevalence and Trends of Developmental Disabilities among Children in the United States: 2009–2017. Pediatrics. 2019;144(4):e20190811. 10.1542/peds.2019-0811. 31558576 10.1542/peds.2019-0811 PMC7076808

[10] Lean RE, Han RH, Smyser TA, Kenley JK, Shimony JS, Rogers CE, et al. Altered neonatal white and gray matter microstructure is associated with neurodevelopmental impairments in very preterm infants with high-grade brain injury. Pediatr Res. 2019;86(3):365–74. 10.1038/s41390-019-0461-1 . PubMed PMID: 31212303. 31212303 10.1038/s41390-019-0461-1 PMC6702093

[11] Srinivasakumar P, Limbrick D, Munro R, Mercer D, Rao R, Inder T, et al. Posthemorrhagic Ventricular Dilatation—Impact on Early Neurodevelopmental Outcome. Am J Perinatol. 2013;30(3):207–14. 10.1055/s-0032-1323581. 22898993 10.1055/s-0032-1323581

[12] Al Rifai MT, Al Tawil KI. The Neurological Outcome of Isolated PVL and Severe IVH in Preterm Infants: Is It Fair to Compare? Pediatr Neurol. 2015;53(5):427–33. 10.1016/j.pediatrneurol.2015.04.004. 26476149 10.1016/j.pediatrneurol.2015.04.004

[13] Wang S, Fan G, Xu K, Wang C. Potential of diffusion tensor MR imaging in the assessment of cognitive impairments in children with periventricular leukomalacia born preterm. Eur J Radiol. 2013;Special Section: Imaging of the Peripheral Nervous System82(1):158–64. 10.1016/j.ejrad.2012.06.032 10.1016/j.ejrad.2012.06.032 23084875

[14] Bolisetty S, Dhawan A, Abdel-Latif M, Bajuk B, Stack J, Lui K, et al. Intraventricular Hemorrhage and Neurodevelopmental Outcomes in Extreme Preterm Infants. Pediatrics. 2014;133(1):55–62. 10.1542/peds.2013-0372 . PubMed PMID: 24379238. 24379238 10.1542/peds.2013-0372

[15] Diamond A. Executive Functions. Annu Rev Psychol. 2013;64(1):135–68. 10.1146/annurev-psych-113011. -143750 PubMed PMID: 23020641. 23020641 10.1146/annurev-psych-113011-143750 PMC4084861

[16] Brydges CR, Landes JK, Reid CL, Campbell C, French N, Anderson M. Cognitive outcomes in children and adolescents born very preterm: a meta-analysis. Dev Med Child Neurol. 2018;60(5):452–68. 10.1111/dmcn.13685. 29453812 10.1111/dmcn.13685

[17] van Houdt CA, Oosterlaan J, van Wassenaer-Leemhuis AG, van Kaam AH, Aarnoudse‐Moens CSH. Executive function deficits in children born preterm or at low birthweight: a meta-analysis. Dev Med Child Neurol. 2019;61(9):1015–24. 10.1111/dmcn.14213. 30945271 10.1111/dmcn.14213 PMC6850293

[18] Mulder H, Pitchford NJ, Hagger MS, Marlow N. Development of Executive Function and Attention in Preterm Children: A Systematic Review. Dev Neuropsychol. 2009;34(4):393–421. 10.1080/87565640902964524. 20183707 10.1080/87565640902964524

[19] Aarnoudse-Moens CSH, Duivenvoorden HJ, Weisglas-Kuperus N, Van Goudoever JB, Oosterlaan J. The profile of executive function in very preterm children at 4 to 12 years. Dev Med Child Neurol. 2012;54(3):247–53. 10.1111/j.1469-8749.2011.04150.x. 22126188 10.1111/j.1469-8749.2011.04150.x

[20] Aarnoudse-Moens CSH, Smidts DP, Oosterlaan J, Duivenvoorden HJ, Weisglas-Kuperus N. Executive Function in Very Preterm Children at Early School Age. J Abnorm Child Psychol. 2009;37(7):981–93. 10.1007/s10802-009-9327-z. 19488851 10.1007/s10802-009-9327-z PMC2734253

[21] Lean RE, Gerstein ED, Smyser TA, Smyser CD, Rogers CE. Socioeconomic disadvantage and parental mood/affective problems links negative parenting and executive dysfunction in children born very preterm. Dev Psychopathol. 2023;35(3):1092–107. 0954579421000961 PubMed PMID: 34725016; PubMed Central PMCID: PMC9058043. 34725016 10.1017/S0954579421000961 PMC9058043

[22] Wehrle FM, Kaufmann L, Benz LD, Huber R, O’Gorman RL, Latal B, et al. Very preterm adolescents show impaired performance with increasing demands in executive function tasks. Early Hum Dev. 2016;92:37–43. 10.1016/j.earlhumdev.2015.10.021. 26651084 10.1016/j.earlhumdev.2015.10.021

[23] Lee SJ, Woodward LJ, Moor S, Austin NC. Executive functioning challenges of adolescents born extremely and very preterm. Front Psychol. 2024;15. 10.3389/fpsyg.2024.1487908. 10.3389/fpsyg.2024.1487908 PMC11669177 39723405

[24] Edgin JO, Inder TE, Anderson PJ, Hood KM, Clark CA, Woodward LJ. Executive functioning in preschool children born very preterm: relationship with early white matter pathology. J Int Neuropsychol Soc. 2008;14(01):90–101. 18078535 10.1017/S1355617708080053

[25] Burnett AC, Scratch SE, Lee KJ, Cheong J, Searle K, Hutchinson E, et al. Executive Function in Adolescents Born < 1000 g or < 28 Weeks: A Prospective Cohort Study. Pediatrics. 2015;135(4):e826–34. 10.1542/peds.2014-3188. 25802342 10.1542/peds.2014-3188

[26] Lee SW, Guo NW, Huang CC, Huang PC, Chiang CJ, Chien YH. Development of cool and hot executive function deficit in children born very low birth weight with normal early development: A longitudinal cohort from aged 6 to 10. Early Hum Dev. 2022;175:105693. 10.1016/j.earlhumdev.2022.105693. 36436312 10.1016/j.earlhumdev.2022.105693

[27] Stålnacke J, Lundequist A, Böhm B, Forssberg H, Smedler AC. A longitudinal model of executive function development from birth through adolescence in children born very or extremely preterm. Child Neuropsychol. 2019;25(3):318–35. 10.1080/09297049.2018.1477928 . PubMed PMID: 29847202. 29847202 10.1080/09297049.2018.1477928

[28] Everts R, Schöne CG, Mürner-Lavanchy I, Steinlin M. Development of executive functions from childhood to adolescence in very preterm-born individuals - A longitudinal study. Early Hum Dev. 2019;129:45–51. 10.1016/j.earlhumdev.2018.12.012. 30639465 10.1016/j.earlhumdev.2018.12.012

[29] Luu TM, Vohr BR, Allan W, Schneider KC, Ment LR. Evidence for Catch-up in Cognition and Receptive Vocabulary Among Adolescents Born Very Preterm. Pediatrics. 2011;128(2):313–22. 10.1542/peds.2010-2655. 21768322 10.1542/peds.2010-2655 PMC3146356

[30] Fitzallen GC, Taylor HG, Bora S. What Do We Know About the Preterm Behavioral Phenotype? A Narrative Review. Front Psychiatry. 2020;11. 10.3389/fpsyt.2020.00154. PubMed PMID: 32269532; PubMed Central PMCID: PMC7109291. 10.3389/fpsyt.2020.00154 PMC7109291 32269532

[31] White LK, Moore TM, Calkins ME, Wolf DH, Satterthwaite TD, Leibenluft E, et al. An Evaluation of the Specificity of Executive Function Impairment in Developmental Psychopathology. J Am Acad Child Adolesc Psychiatry. 2017;56(11):975–e9823. 10.1016/j.jaac.2017.08.016. 29096780 10.1016/j.jaac.2017.08.016 PMC5815390

[32] Yang Y, Shields GS, Zhang Y, Wu H, Chen H, Romer AL. Child executive function and future externalizing and internalizing problems: A meta-analysis of prospective longitudinal studies. Clin Psychol Rev. 2022;97:102194. 10.1016/j.cpr.2022.102194 . PubMed PMID: 35964337. 35964337 10.1016/j.cpr.2022.102194

[33] Zelazo PD. Executive Function and Psychopathology: A Neurodevelopmental Perspective. Annu Rev Clin Psychol. 2020;16(1):431–54. 10.1146/annurev-clinpsy-072319-024242 . PubMed PMID: 32075434. 32075434 10.1146/annurev-clinpsy-072319-024242

[34] Demetriou EA, Lampit A, Quintana DS, Naismith SL, Song YJC, Pye JE, et al. Autism spectrum disorders: a meta-analysis of executive function. Mol Psychiatry. 2018;23(5):1198–204. 10.1038/mp.2017.75. 28439105 10.1038/mp.2017.75 PMC5984099

[35] Zhou Q, Chen SH, Main A. Commonalities and Differences in the Research on Children’s Effortful Control and Executive Function: A Call for an Integrated Model of Self-Regulation: Effortful Control and Executive Function. Child Dev Perspect. 2012;6(2):112–21. 10.1111/j.1750-8606.2011.00176.x.

[36] Bridgett DJ, Oddi KB, Laake LM, Murdock KW, Bachmann MN. Integrating and differentiating aspects of self-regulation: Effortful control, executive functioning, and links to negative affectivity. Emotion. 2013;13(1):47–63. 10.1037/a0029536 . PubMed PMID: 22906086. 22906086 10.1037/a0029536

[37] Rinsky JR, Hinshaw SP. Linkages between childhood executive functioning and adolescent social functioning and psychopathology in girls with ADHD. Child Neuropsychol. 2011;17(4):368–90. .544649 PubMed PMID: 21390921. 21390921 10.1080/09297049.2010.544649 PMC3120930

[38] Wade M, Zeanah CH, Fox NA, Nelson CA. Global deficits in executive functioning are transdiagnostic mediators between severe childhood neglect and psychopathology in adolescence. Psychol Med. 2020;50(10):1687–94. 0033291719001764 PubMed PMID: 31391139 PMCID: PMC8026012. 31391139 10.1017/S0033291719001764 PMC8026012

[39] Meyer SE, Carlson GA, Wiggs EA, Martinez PE, Ronsaville DS, Klimes–Dougan B, et al. A prospective study of the association among impaired executive functioning, childhood attentional problems, and the development of bipolar disorder. Dev Psychopathol. 2004;16(2):461–76. doi:10.1017/S095457940404461X PubMed PMID: 15487606. 15487606 10.1017/s095457940404461x

[40] Orm S, Andersen PN, Teicher MH, Fossum IN, Øie MG, Skogli EW. Childhood executive functions and ADHD symptoms predict psychopathology symptoms in emerging adults with and without ADHD: a 10-year longitudinal study. Res Child Adolesc Psychopathol. 2023;51(2):261–71. 10.1007/s10802-022-00957-7 . PubMed PMID: 36194356 PMCID: PMC9867664. 36194356 10.1007/s10802-022-00957-7 PMC9867664

[41] Alduncin N, Huffman LC, Feldman HM, Loe IM. Executive function is associated with social competence in preschool-aged children born preterm or full term. Early Hum Dev. 2014;90(6):299–306. 10.1016/j.earlhumdev.2014.02.011. 24661446 10.1016/j.earlhumdev.2014.02.011 PMC4240273

[42] Korzeniewski SJ, Joseph RM, Kim SH, Allred EN, O’Shea TM, Leviton A, et al. Social Responsiveness Scale Assessment of the Preterm Behavioral Phenotype in Ten-Year-Olds Born Extremely Preterm. J Dev Behav Pediatr JDBP. 2017;38(9):697–705. .0000000000000485 PubMed PMID: 28857804; PubMed Central PMCID: PMC5668158. 28857804 10.1097/DBP.0000000000000485 PMC5668158

[43] Wolfe KR, Vannatta K, Nelin MA, Yeates KO. Executive functions, social information processing, and social adjustment in young children born with very low birth weight. Child Neuropsychol. 2015;21(1):41–54. PubMed PMID: 24344821. 24344821 10.1080/09297049.2013.866217

[44] Montagna A, Karolis V, Batalle D, Counsell S, Rutherford M, Arulkumaran S, et al. ADHD symptoms and their neurodevelopmental correlates in children born very preterm. PLoS ONE. 2020;15(3). 10.1371/journal.pone.0224343 . PubMed PMID: 32126073; PubMed Central PMCID: PMC7053718. 10.1371/journal.pone.0224343 PMC7053718 32126073

[45] Schnider B, Disselhoff V, Held U, Latal B, Hagmann CF, Wehrle FM. Executive function deficits mediate the association between very preterm birth and behavioral problems at school-age. Early Hum Dev. 2020;146:105076. 10.1016/j.earlhumdev.2020.105076. 32470766 10.1016/j.earlhumdev.2020.105076

[46] Twilhaar ES, de Kieviet JF, Bergwerff CE, Finken MJJ, van Elburg RM, Oosterlaan J. Social Adjustment in Adolescents Born Very Preterm: Evidence for a Cognitive Basis of Social Problems. J Pediatr. 2019;213:66–e731. 10.1016/j.jpeds.2019.06.045. 31402139 10.1016/j.jpeds.2019.06.045

[47] Papile LA, Burstein J, Burstein R, Koffler H. Incidence and evolution of subependymal and intraventricular hemorrhage: A study of infants with birth weights less than 1,500 gm. J Pediatr. 1978;92(4):529–34. 10.1016/S0022-3476(78)80282-0. 305471 10.1016/s0022-3476(78)80282-0

[48] Wellons JC, Shannon CN, Holubkov R, Riva-Cambrin J, Kulkarni AV, Limbrick DD, et al. Shunting outcomes in posthemorrhagic hydrocephalus: results of a Hydrocephalus Clinical Research Network prospective cohort study. J Neurosurg Pediatr. 2017;20(1):19–29. 10.3171/2017.1.PEDS16496. 28452657 10.3171/2017.1.PEDS16496

[49] Cyr PEP, Lean RE, Kenley JK, Kaplan S, Meyer DE, Neil JJ, et al. Neonatal motor functional connectivity and motor outcomes at age two years in very preterm children with and without high-grade brain injury. NeuroImage Clin. 2022;36:103260. 10.1016/j.nicl.2022.103260 . PubMed PMID: 36451363; PubMed Central PMCID: PMC9668638. 36451363 10.1016/j.nicl.2022.103260 PMC9668638

[50] Elliott CD. Differential Ability Scales. Second Edition. San Antonio, TX: Harcourt Assessment; 2007.

[51] Espy KA, Bull R, Martin J, Stroup W. Measuring the development of executive control with the shape school. Psychol Assess. 2006;18(4):373–81. 10.1037/1040-3590.18.4.373. Located at: 2006-22005-002. 17154758 10.1037/1040-3590.18.4.373

[52] National Institutes of Health Toolbox. Cognition Battery (NIH Toolbox CB). Monogr Soc Res Child Dev. 2013;78(4):1–172. 10.1111/mono.1203. 10.1111/mono.12044 23952209

[53] Brydges CR, Reid CL, Fox AM, Anderson M. A unitary executive function predicts intelligence in children. Intelligence. 2012;40(5):458–69. 10.1016/j.intell.2012.05.006.

[54] McKenna R, Rushe T, Woodcock KA. Informing the Structure of Executive Function in Children: A Meta-Analysis of Functional Neuroimaging Data. Front Hum Neurosci. 2017;11(154):Published Online. 10.3389/fnhum.2017.00154 10.3389/fnhum.2017.00154 PMC5383671 28439231

[55] Willoughby M, Blair C. Test-retest reliability of a new executive function battery for use in early childhood. Child Neuropsychol. 2011;17(6):564–79. 10.1080/09297049.2011.554390 . PubMed PMID: 21714751. 21714751 10.1080/09297049.2011.554390

[56] Friedman NP, Banich MT. Questionnaires and task-based measures assess different aspects of self-regulation: Both are needed. Proc Natl Acad Sci. 2019;116(49):24396–7. 10.1073/pnas.1915315116. 31719199 10.1073/pnas.1915315116 PMC6900513

[57] Achenbach TM, Rescorla LA. Manual for the ASEBA School-Age Forms & Profiles. Burlington, VT: University of Vermont, Research Center for Children, Youth, & Families; 2001.

[58] Birmaher B, Khrtarpal S, Brent D, Cully M, Balach L, Kaufam J, et al. The Screen for Child Anxiety Related Emotional Disorders (SCARED): Scale Construction and Psychometric Characteristics. J Am Acad Child Adolesc Psychiatry. 1997;36(4):545–53. 10.1097/00004583-199704000-00018. 9100430 10.1097/00004583-199704000-00018

[59] Constantino JN, Gruber CP. Social Responsiveness Scale, Second Edition (SRS-2). Los Angeles, CA: Western Psychological Services; 2012.

[60] Conners C. Conners 3rd Edition. New York: Multi-Health Systems Inc.; 2007.

[61] Achenbach TM, McConaughy SH, Ivanova MY, Rescorla LA. Manual for the ASEBA Brief Problem Monitor^™^ for Ages 6–18 (BPM/6–18). University of Vermont: Resarch Center for Children, Youth, and Families; 2017.

[62] Brooks BL, Sherman EMS, Strauss E, NEPSY-II:. A Developmental Neuropsychological Assessment, Second Edition. Child Neuropsychol. 2009;16(1):80–101. 10.1080/09297040903146966

[63] Bishop CL, Lean RE, Smyser TA, Smyser CD, Rogers CE. Adverse Childhood Experiences and Socioemotional Outcomes of Children Born Very Preterm. J Pediatr. 2025;276. 10.1016/j.jpeds.2024.114377 . PubMed PMID: 39442792. 10.1016/j.jpeds.2024.114377 PMC11851865 39442792

[64] Carter AS, Briggs-Gowan MJ, Jones SM, Little TD. The Infant-Toddler Social and Emotional Assessment (ITSEA): factor structure, reliability, and validity. J Abnorm Child Psychol. 2003;31(5):495–514. PubMed PMID: 14561058. 14561058 10.1023/a:1025449031360

[65] Wechsler D. WPPSI-III: Administration and scoring manual. San Antonio, TX: The Psychological Corporation; 2004.

[66] Wechsler D, Hsiao-pin C. WASI-II: Wechsler abbreviated scale of intelligence. San Antonio, TX: Pearson; 2011.

[67] Wechsler D. Wechsler Test of Adult Reading. San Antonio, TX: The Psychological Corporation; 2001.

[68] Kind AJH, Buckingham WR, Making Neighborhood-Disadvantage Metrics Accessible - The Neighborhood Atlas. N Engl J Med. 2018;378(26):2456–8. doi:10.1056/NEJMp1802313 PubMed PMID: 29949490; PubMed Central PMCID: PMC6051533. 29949490 10.1056/NEJMp1802313 PMC6051533

[69] Beck AT, Steer RA, Brown GK. Manual for the Beck Depression Inventory-II. San Antonio, TX: Psychological Corporation; 1996.

[70] Spielberger CD, Gorsuch RL, Lushene R, Vagg PR, Jacobs GA. Manual for the State-Trait Anxiety Inventory. Palo Alto, CA: Consulting Psychology; 1983.

[71] Abidin RR. Parenting Stress Index (PSI). Charlottesville, VA: Pediatric Psychology Stress; 1990.

[72] Sarason IG, Levine HM, Basham RB, Sarason BR. Assessing social support: The Social Support Questionnaire. J Pers Soc Psychol. 1983;44(1):127–39. 10.1037/0022-3514.44.1.127.

[73] Romer AL, Pizzagalli DA. Is executive dysfunction a risk marker or consequence of psychopathology? A test of executive function as a prospective predictor and outcome of general psychopathology in the adolescent brain cognitive development study^®^. Dev Cogn Neurosci. 2021;51:100994. 10.1016/j.dcn.2021.100994 . PubMed PMID: 34332330 PMCID: PMC8340137. 34332330 10.1016/j.dcn.2021.100994 PMC8340137

[74] Castro-Schilo L, Grimm KJ. Using residualized change versus difference scores for longitudinal research. J Soc Pers Relatsh. 2018;35(1):32–58. 10.1177/0265407517718387.

[75] Twisk J, Proper K. Analysis of covariance vs. residual change. J Clin Epidemiol. 2005;58(5):542. 10.1016/j.jclinepi.2004.12.003. 15845342 10.1016/j.jclinepi.2004.12.002

[76] Hayes A. Beyond Baron and Kenny: Statistical Mediation Analysis in the New Millennium. Commun Monogr - COMMUN MONOGR. 2009;76:408–20. 10.1080/03637750903310360.

[77] Pritchard VE, Woodward LJ. Preschool executive control on the Shape School task: Measurement considerations and utility. Psychol Assess. 2011;23(1):31–43. 10.1037/a0021095. 21381841 10.1037/a0021095

[78] Broomell APR, Bell MA. Longitudinal development of executive function from infancy to late childhood. Cogn Dev. 2022;63:101229. 10.1016/j.cogdev.2022.101229. 40746389 10.1016/j.cogdev.2022.101229 PMC12311735

[79] Last BS, Lawson GM, Breiner K, Steinberg L, Farah MJ. Childhood socioeconomic status and executive function in childhood and beyond. PLoS ONE. 2018;13(8):e0202964. 10.1371/journal.pone.0202964 . PubMed PMID: 30142188; PubMed Central PMCID: PMC6108482. 30142188 10.1371/journal.pone.0202964 PMC6108482

[80] Lawson GM, Hook CJ, Hackman DA, Farah MJ. Socioeconomic Status and Neurocognitive Development: Executive Function. In: Griffin JA, Freund LS, McCardle P, editors. Executive Function in Preschool Children: Integrating Measurement, Neurodevelopment, and Translational Research. Washington (DC): American Psychological Association; 2016. p. 28.

[81] Böhm B, Katz-Salamon M, Smedler AC, Lagercrantz H, Forssberg H. Developmental risks and protective factors for influencing cognitive outcome at 5½ years of age in very-low-birthweight children. Dev Med Child Neurol. 2002;44(8):508–16. 10.1111/j.1469-8749.2002.tb00321.x. 12206615 10.1017/s001216220100247x

[82] Woodward LJ, Clark CAC, Pritchard VE, Anderson PJ, Inder TE. Neonatal White Matter Abnormalities Predict Global Executive Function Impairment in Children Born Very Preterm. Dev Neuropsychol. 2011;36(1):22–41. 10.1080/87565641.2011.540530. 21253989 10.1080/87565641.2011.540530

[83] Diamond A, Lee K. Interventions Shown to Aid Executive Function Development in Children 4 to 12 Years Old. Science. 2011;333(6045):959–64. 10.1126/science.1204529 . PubMed PMID: 21852486 PMCID: PMC3159917. 21852486 10.1126/science.1204529 PMC3159917

[84] Spittle A, Orton J, Anderson PJ, Boyd R, Doyle LW. Early developmental intervention programmes provided post hospital discharge to prevent motor and cognitive impairment in preterm infants - Spittle, A – 2015 | Cochrane Library. Available from: https://www.cochranelibrary.com/cdsr/doi/10.1002/14651858.CD005495.pub4/full. [Cited 2025 Oct 19]. 10.1002/14651858.CD005495.pub4 PMC8612699 26597166

[85] Ku S, Feng X. Maternal depressive symptoms and the growth of child executive function: Mediation by maternal sensitivity. J Fam Psychol. 2023;37(4):421–31. 10.1037/fam0000832. 33661684 10.1037/fam0000832

[86] Nadeau L, Tessier R, Boivin M, Lefebvre F, Robaey P. Extremely Premature and Very Low Birthweight Infants: A Double Hazard Population? Soc Dev. 2003;12(2):235–48. 10.1111/1467-9507.00231.

[87] Woodward LJ, Lu Z, Morris AR, Healey DM. Preschool self regulation predicts later mental health and educational achievement in very preterm and typically developing children. Clin Neuropsychol. 2017;31(2):404–22. 10.1080/13854046.2016.1251614 . PubMed PMID: 27801620. 27801620 10.1080/13854046.2016.1251614

[88] Taylor HG. Neurodevelopmental origins of social competence in very preterm children. Semin Fetal Neonatal Med. 2020;Long term outcomes following very preterm birth25(3):101108. 10.1016/j.siny.2020.101108 10.1016/j.siny.2020.101108 PMC7363558 32284233

[89] Marleau I, Vona M, Gagner C, Luu TM, Beauchamp MH. Social cognition, adaptive functioning, and behavior problems in preschoolers born extremely preterm. Child Neuropsychol. 2021;27(1):96–108. 2020.1797656 PubMed PMID: 32716689. 32716689 10.1080/09297049.2020.1797656

[90] Montagna A, Nosarti C. Socio-Emotional Development Following Very Preterm Birth: Pathways to Psychopathology. Front Psychol. 2016;7. 10.3389/fpsyg.2016.00080. PubMed PMID: 26903895; PubMed Central PMCID: PMC4751757. 10.3389/fpsyg.2016.00080 PMC4751757 26903895

[91] Faure N, Habersaat S, Harari MM, Müller-Nix C, Borghini A, Ansermet F, et al. Maternal Sensitivity: a Resilience Factor against Internalizing Symptoms in Early Adolescents Born Very Preterm? J Abnorm Child Psychol. 2017;45(4):671–80. 10.1007/s10802-016-0194-0. 27573689 10.1007/s10802-016-0194-0

[92] Samuelsson M, Holsti A, Adamsson M, Serenius F, Hägglöf B, Farooqi A. Behavioral Patterns in Adolescents Born at 23 to 25 Weeks of Gestation. Pediatrics. 2017;e20170199. 10.1542/peds.2017-0199 . PubMed PMID: 28642374. 10.1542/peds.2017-0199 28642374

[93] Bhutta AT, Cleves MA, Casey PH, Cradock MM, Anand KJS. Cognitive and Behavioral Outcomes of School-Aged Children Who Were Born PretermA Meta-analysis. JAMA. 2002;288(6):728–37. 10.1001/jama.288.6.728. 12169077 10.1001/jama.288.6.728

[94] Ge MW, Shen LT, Attiq UR, Li W, Du W, Peng XY, et al. Internalizing and Externalizing Symptoms in Children and Adolescents Born Preterm or Low Birth Weight: A Meta-Analysis. J Youth Adolesc. 2025;54(10):2660–80. 10.1007/s10964-025-02229-1. 40739415 10.1007/s10964-025-02229-1

[95] Hill EL. Evaluating the theory of executive dysfunction in autism. Dev Rev. 2004;24(2):189–233. 10.1016/j.dr.2004.01.001.

[96] Mutter B, Alcorn MB, Welsh M. Theory of mind and executive function: working-memory capacity and inhibitory control as predictors of false-belief task performance. Percept Mot Skills. 2006;102(3):819–35. 10.2466/pms.102.3.819-835 . PubMed PMID: 16916162. 16916162 10.2466/pms.102.3.819-835

[97] Lecce S, Bianco F. Working memory predicts changes in children’s theory of mind during middle childhood: A training study. Cogn Dev. 2018;47:71–81. 10.1016/j.cogdev.2018.04.002.

[98] Moeller J. A word on standardization in longitudinal studies: don’t. Front Psychol. 2015;6:1389. 10.3389/fpsyg. 2015.01389 PubMed PMID: 26441764; PubMed Central PMCID: PMC4569815. 26441764 10.3389/fpsyg.2015.01389 PMC4569815

[99] Jester JM, Nigg JT, Puttler LI, Long JC, Fitzgerald HE, Zucker RA. Intergenerational transmission of neuropsychological executive functioning. Brain Cogn. 2009;70(1):145–53. 10.1016/j.bandc.2009.01.005 . PubMed PMID: 19243871 PMCID: PMC2680419. 19243871 10.1016/j.bandc.2009.01.005 PMC2680419

