# Supplementary material for: Executive function and implications for the Preterm Behavioral Phenotype in very preterm children at age 9–10 years
Source: J Neurodev Disord. 2026 May 28;18:45. doi: 10.1186/s11689-026-09710-3 (PMC13412151; doi:10.1186/s11689-026-09710-3)
Supplement: Supplementary file 1 — Supplementary Material 1: Table S1. Summary of tasks and questionnaires used to assess key domains of interest. Table S2. Bivariate correlations between Infant and Toddler Social and Emotional Assessment (ITSEA) problems at age 2 years and Preterm Behavioral Phenotype (PBP) outcomes with significant between-groups differences at age 9–10 years (n = 202). Table S3. Bivariate correlations between socioenvironmental factors in early childhood and executive function (EF) outcomes at ages 5 and 9–10 years (n = 202). Table S4. Summary of linear-mixed effects model additionally including maternal IQ as a predictor of global executive function outcome at age 9–10 years (n = 202). Table S5. Summary of linear-mixed effects model relating group, short-term memory at age 5 years, and maternal and family factors at the 5-year follow-up to working memory outcome at age 9–10 years (n = 202). Table S6. Summary of linear-mixed effects model relating group, inhibitory control at age 5 years, and maternal and family factors at the 5-year follow-up to inhibitory control outcome at age 9–10 years (n = 202). Table S7. Summary of linear-mixed effects model relating group, shifting/flexibility at age 5 years, and maternal and family factors at the 5-year follow-up to shifting/flexibility outcome at age 9–10 years (n = 202). Table S8. Bivariate correlations between infant clinical factors and primary outcome measures among the VPT and WMI groups. Table S9. Associations between infant clinical factors and primary outcome measures among the VPT and WMI groups after accounting for group and covariate factors. Table S10. Summary of serial mediation analysis testing global executive function (EF) as a mediator between group and Preterm Behavioral Phenotype (PBP) outcomes with additional adjustment for infant socio-emotional problems at age 2 years (n = 202). Table S11. Summary of serial mediation analysis testing short-term/working memory (WM) as a mediator between group and Preterm Behavioral P [file 11689_2026_9710_MOESM1_ESM.docx]

**SUPPLEMENTARY MATERIAL FOR**

**Executive function and implications for the Preterm Behavioral Phenotype in Very Preterm Children at age 9-10 Years**

**Rachel E. Lean^1^, Berenice Anya^1^, Lisa Gorham^1^, Christopher D. Smyser^2,3,4^, Cynthia E. Rogers^1,2^**

^1^ Department of Psychiatry, Washington University School of Medicine, St. Louis, MO 63110

^2^ Department of Pediatrics, Washington University School of Medicine, St. Louis, MO 63110

^3^ Department of Radiology, Washington University School of Medicine, St. Louis, MO 63110

^4^ Department of Neurology, Washington University School of Medicine, St. Louis, MO 63110

**Correspondence to:** Dr. R.E. Lean. Washington University School of Medicine, Campus Box 8514, St. Louis, MO 63110. Phone: (314) 23-0854. E-mail: rachel.lean@wustl.edu

**TABLES**

| **Table S1.** Summary of tasks and questionnaires used to assess key domains of interest. | | | |
| --- | --- | --- | --- |
| **Executive Function (EF)** | | |  |
| **Domain** | **Age 5 Years** | **Age 9-10 Years** | **Bivariate correlation ^a^** |
| Short-term/working memory | DAS Digits Forward | NIHTB List Sort Working Memory | .40*** |
| Inhibitory control | Shape School Inhibit condition | NIHTB Flanker Task of Inhibitory Control and Attention | .20* |
| Shifting/flexibility | Shape School Shift condition | NIHTB Dimensional Card Sort Task | .38*** |
| **Preterm Behavioral Phenotype (PBP) at age 9-10 years** | | |  |
| **Domain** | **Parent-Informant** | **Child-Informant** | **Bivariate correlation ^a^** |
| Internalizing/anxiety problems | CBCL/6-18 Internalizing subscale | BPM-Y Internalizing subscale | .19* |
|  | SCARED total score |  | .22** |
| Social communication-interaction differences | SRS-2 total problems | NEPSY-II Social Perception: Affect recognition subtest | -.29*** |
|  |  | NEPSY-II Social Perception: Theory of mind subtest | -.45*** |
| Attention deficit/hyperactivity symptoms | Conners’ 3 Inattention subscale | BPM-Y Attention Problems subscale | .22** |
|  | Conners’ 3 Hyperactivity subscale |  | .21** |
| *Note.* DAS, Differential Ability Scale (1); NIHTB, National Institutes of Health Toolbox Cognitive Battery (2); CBCL/6-18, Child Behavior Checklist 6-18 (3); SCARED, Screen for Anxiety Related Disorders (4); SRS-2, Social Responsiveness Scale-2 (5); BPM-Y, Brief Problem Monitor-Youth (6); NEPSY-II, Developmental Neuropsychological Assessment-II (7).  ^a^ Correlations reported in complete cases.  * *p* ≤ .05, ** *p* ≤ .01, *** *p* ≤ .001 | | | |

| **Table S2.** Bivariate correlations between Infant and Toddler Social and Emotional Assessment (ITSEA) problems at age 2 years and Preterm Behavioral Phenotype (PBP) outcomes with significant between-groups differences at age 9-10 years (*n* = 202). | | | |
| --- | --- | --- | --- |
| **ITSEA Subscales** | **Parent-informant social communication-interaction differences** | **Child-informant PBP composite score** | **Child-informant theory of mind** **^a^** |
| Externalizing | 0.31^**^ | 0.09 | 0.003 |
| Internalizing | 0.07 | 0.02 | -0.04 |
| Dysregulation | 0.28^**^ | 0.25^*^ | -0.24^*^ |
| Social Competency ^a^ | -0.42*** | -0.34^***^ | 0.35^***^ |
| ^a^ Higher scores = better outcome  ** p* < .05, ** *p* < .01, *** *p* < .001 | | | |

As shown in Table S2, ITSEA (8) dysregulation and social competency problem ratings were correlated with all three PBP outcomes of interest that had significant between-groups differences at age 9-10 years (*p*<.05, Table 4, main manuscript). ITSEA dysregulation and social competency scales were, therefore, included as additional covariates in serial mediation analysis (see Table S10) to account for early socioemotional problems assessed prior to EF and PBP outcomes.

| **Table S3.** Bivariate correlations between socioenvironmental factors in early childhood and executive function (EF) outcomes at ages 5 and 9-10 years (*n* = 202) | | | | | | | | |
| --- | --- | --- | --- | --- | --- | --- | --- | --- |
| **Socioenvironmental factors at age 5 years**  *Domain (measure)* | **EF at age 5 Years** | | | | **EF at age 9-10 years** | | | |
|  | **Global EF** | **Short-term memory** | **Inhibitory control** | **Shifting** | **Global EF** | **Working memory** | **Inhibitory control** | **Shifting** |
| Social Disadvantage composite ^a^ | -0.34^***^ | -0.29^***^ | -0.19^*^ | -0.19 | -0.39^***^ | -0.33^***^ | -0.29^***^ | -0.37^***^ |
| Maternal depression symptoms (BDI) | -0.15 | -0.16 | -0.07 | -0.06 | -0.22^**^ | -0.21^*^ | -0.17^*^ | -0.15 |
| Maternal trait anxiety problems (STAI) | -0.15 | -0.11 | -0.10 | -0.08 | -0.07 | -0.07 | -0.08 | -0.03 |
| Stressful/traumatic life events (PSI) | -0.26^**^ | -0.25^**^ | -0.15 | -0.11 | -.19^*^ | -0.17 | -0.13 | -0.15 |
| Parenting stress (PSI) | -0.09 | -0.14 | -0.05 | 0.02 | -0.11 | -0.04 | -0.13 | -0.10 |
| General family dysfunction (FAD) | -0.08 | -0.06 | -0.08 | -0.02 | -0.07 | -0.04 | -0.06 | -0.06 |
| Observed non-supportive parenting behavior ^b^ (PCIRS) | -0.21^*^ | -0.14 | -0.10 | -0.16 | -0.20 | -0.15 | -0.10 | -0.22^*^ |
| Observed supportive parenting behavior ^c^ (PCIRS) | 0.04 | 0.10 | -0.03 | 0.01 | -0.003 | -0.03 | -0.03 | 0.06 |
| Cognitive stimulation provided in the home (Stim-Q) | 0.15 | 0.24^*^ | -0.02 | 0.09 | 0.07 | 0.12 | -0.04 | 0.07 |
| Maternal FSIQ (WTAR) | 0.22^*^ | 0.23^*^ | 0.12 | 0.08 | 0.28^**^ | 0.28^**^ | 0.14 | 0.24^**^ |
| *Note.* Measures: BDI, Beck Depression Inventory (9); STAI, State Trait Anxiety Inventory (10); PSI, Parent Stress Index (11); FAD, McMaster Family Assessment Device (12); PCIRS, Parent Child Interaction Rating Scales (13); Stim-Q (14); WTAR, Wechsler Test of Adult Reading (15). FSIQ, Full scale intelligence quotient.  ^a^ Composite of maternal demographic stressor index, family income-to-needs ratio, neighborhood Area Deprivation Index (16)  ^b^ Composite of intrusiveness and negative regard (17)  ^c^ Composite of sensitivity, positive regard, and stimulation of cognition (17)  ** p* < .05, ** *p* < .01, *** *p* < .001 | | | | | | | | |

| **Table S4.** Summary of linear-mixed effects model additionally including maternal IQ as a predictor of global executive function outcome at age 9-10 years (*n* = 202). | | | | | | |
| --- | --- | --- | --- | --- | --- | --- |
| **Independent variables** | **Estimate** | **SE** | ***t*** | ***p*** | **95% Confidence interval** | |
|  |  |  |  |  | **Lower** | **Upper** |
| Group ^a^ |  |  |  |  |  |  |
| *WMI* | -0.82 | 0.17 | -4.88 | <.001 | -1.15 | -0.49 |
| *VPT* | -0.35 | 0.14 | -2.53 | .01 | -0.62 | -0.08 |
| Global EF at age 5 years | 0.23 | 0.11 | 2.12 | .04 | 0.01 | 0.44 |
| Social disadvantage composite at age 5 years | -0.24 | 0.12 | -2.09 | .04 | -0.47 | -0.01 |
| Maternal depression symptoms at age 5 years | -0.01 | 0.01 | -1.57 | .12 | -0.03 | 0.004 |
| Stressful/traumatic life events at age 5 years | 0.001 | 0.01 | -0.05 | .96 | -0.02 | 0.01 |
| Maternal FSIQ | 0.01 | 0.01 | 0.87 | .39 | -0.01 | 0.02 |
| ^a^ Intercepts for WMI and VPT groups are relative to the FT group. | | | | | | |

| **Table S5.** Summary of linear-mixed effects model relating group, short-term memory at age 5 years, and maternal and family factors at the 5-year follow-up to working memory outcome at age 9-10 years (*n* = 202). | | | | | | |
| --- | --- | --- | --- | --- | --- | --- |
| **Independent variables** | **Estimate** | **SE** | ***t*** | ***p*** | **95% Confidence interval** | |
|  |  |  |  |  | **Lower** | **Upper** |
| Group ^a^ |  |  |  |  |  |  |
| *WMI* | -11.10 | 3.46 | -3.20 | .001 | -17.91 | -4.29 |
| *VPT* | -5.51 | 3.05 | -1.81 | .07 | -11.52 | 0.50 |
| Short-term memory at age 5 years | 0.22 | 0.16 | 1.37 | .18 | -0.10 | 0.54 |
| Social disadvantage composite at age 5 years | -4.92 | 1.60 | -3.08 | .002 | -8.08 | -0.77 |
| Maternal depression symptoms at age 5 years | -0.29 | 0.20 | -1.45 | .15 | -0.70 | 0.11 |
| ^a^ Intercepts for WMI and VPT groups are relative to the FT group. | | | | | | |

| **Table S6.** Summary of linear-mixed effects model relating group, inhibitory control at age 5 years, and maternal and family factors at the 5-year follow-up to inhibitory control outcome at age 9-10 years (*n* = 202). | | | | | | |
| --- | --- | --- | --- | --- | --- | --- |
| **Independent variables** | **Estimate** | **SE** | ***t*** | ***p*** | **95% Confidence interval** | |
|  |  |  |  |  | **Lower** | **Upper** |
| Group ^a^ |  |  |  |  |  |  |
| *WMI* | -11.01 | 2.20 | -5.01 | <.001 | -15.33 | -6.69 |
| *VPT* | -4.22 | 1.79 | -2.35 | .02 | -7.74 | -0.70 |
| Inhibitory control at age 5 years | 0.51 | 3.44 | 0.15 | .88 | -6.58 | 7.60 |
| Social disadvantage composite at age 5 years | -3.00 | 1.04 | -2.88 | .005 | -5.06 | -0.94 |
| Maternal depression symptoms at age 5 years | -0.16 | 0.12 | -1.26 | .21 | -0.40 | 0.09 |
| ^a^ Intercepts for WMI and VPT groups are relative to the FT group. | | | | | | |

| **Table S7.** Summary of linear-mixed effects model relating group, shifting/flexibility at age 5 years, and maternal and family factors at the 5-year follow-up to shifting/flexibility outcome at age 9-10 years (*n* = 202). | | | | | | |
| --- | --- | --- | --- | --- | --- | --- |
| **Independent variables** | **Estimate** | **SE** | ***t*** | ***p*** | **95% Confidence interval** | |
|  |  |  |  |  | **Lower** | **Upper** |
| Group ^a^ |  |  |  |  |  |  |
| *WMI* | -7.13 | 2.22 | -3.20 | .001 | -11.49 | -2.76 |
| *VPT* | -3.63 | 1.80 | -2.02 | .04 | -7.16 | -0.11 |
| Shifting/flexibility at age 5 years | 6.31 | 4.91 | 1.29 | .21 | -3.96 | 16.57 |
| Social disadvantage composite at age 5 years | -3.29 | 1.21 | -2.73 | .009 | -5.73 | -0.86 |
| Non-supportive parenting behavior at 5 years | -1.60 | 1.02 | -1.57 | .12 | -3.67 | 0.46 |
| ^a^ Intercepts for WMI and VPT groups are relative to the FT group. | | | | | | |

**Consideration of infant clinical factors.**

Table S8 shows the bivariate correlations between infant clinical factors and the primary composite executive function (EF) and Preterm Behavioral Phenotype (PBP) outcomes of interest, restricting the analysis to VPT and WMI children who were all born ≤ 30 weeks gestational age. As shown, lower gestational age and birthweight was correlated with lower EF abilities at age 9-10 years, whereas higher infant medical risk index scores were correlated with higher PBP composite scores across informants at the 9-10-year follow-up. However, these associations were not significant in multivariable models that included group (WMI relative to VPT), social disadvantage, and maternal psychosocial distress as independent variables (Table S9). As such, the key findings of the study remain unchanged.

| **Table S8.** Bivariate correlations between infant clinical factors and primary outcome measures among the VPT and WMI groups. | | | | |
| --- | --- | --- | --- | --- |
|  | **Global EF** | | **PBP composite** | |
|  | **Age 5 years** | **Age 9-10 years** | **Parent-informant** | **Child-informant** |
| Gestational age (weeks) | -0.003 | .22^*^ | -0.04 | -0.04 |
| Birthweight (grams) | 0.02 | .20^*^ | -0.04 | -0.10 |
| Infant medical risk index | 0.02 | -0.13 | .22^*^ | .24^*^ |
| ** p* < .05, ** *p* < .01, *** *p* < .001 | | | | |

| **Table S9.** Associations between infant clinical factors and primary outcome measures among the VPT and WMI groups after accounting for group and covariate factors. | | | | | | |
| --- | --- | --- | --- | --- | --- | --- |
|  | **Global EF at age 9-10 years** | | **Parent-informant PBP composite** | | **Child-informant PBP composite** | |
|  | **Estimate (SE)** | ***p*** | **Estimate (SE)** | ***p*** | **Estimate (SE)** | ***p*** |
| ***Model 1*** |  |  | - | - | - | - |
| Group | -0.43 (0.16) | .008 |  |  |  |  |
| Gestational age | 0.09 (0.05) | .06 |  |  |  |  |
| Social Disadvantage composite | -0.22 (0.08) | .008 |  |  |  |  |
| Maternal psychosocial distress index | -0.17 (0.07) | .02 |  |  |  |  |
| ***Model 2*** |  |  | - | - | - | - |
| Group | -0.52 (0.17) | .002 |  |  |  |  |
| Birthweight | 0.001 (0.00) | .14 |  |  |  |  |
| Social Disadvantage composite | -0.20 (0.08) | .02 |  |  |  |  |
| Maternal psychosocial distress index | -0.17 (0.07) | .02 |  |  |  |  |
| ***Model 3*** |  |  |  |  |  |  |
| Group | - | - | 0.04 (0.34) | .91 | 0.63 (0.30) | .03 |
| Infant medical risk |  |  | 0.12 (0.09) | .20 | 0.10 (0.08) | .19 |
| Social Disadvantage composite |  |  | 0.10 (0.18) | .58 | 0.06 (0.16) | .71 |
| Maternal psychosocial distress index |  |  | 0.70 (0.16) | <.001 | 0.27 (0.17) | .06 |
|  | | | | | | |

| **Table S10.** Summary of serial mediation analysis testing global executive function (EF) as a mediator between group and Preterm Behavioral Phenotype (PBP) outcomes with additional adjustment for infant socio-emotional problems at age 2 years (*n* = 202) | | | | | | |
| --- | --- | --- | --- | --- | --- | --- |
| **Effects:** | **Total effect** | **Direct effect** | **Indirect effect 1****: EF at age 5 years** | **Indirect effect 2: EF at age 9-10 years** | **Indirect effect 3: EF from age 5 to 9-10 years** |  |
|  | *IV → DV* | *IV → DV controlling for M1 and M2* | *IV → M1→ DV* | *IV → M2→ DV* | *IV → M1→ M2 → DV* |  |
| ***Model: Parent-informant social communication-interaction differences mediated by global EF*** | | | | | | **Conclusion** |
| VPT | 0.17 ± 0.12  (-0.07 – 0.40) | 0.05 ± 0.13 (-0.20 – 0.31) | 0.04 ± 0.04 (-0.009 – 0.13) | 0.05 ± 0.03 (0.001 – 0.11) | 0.02 ± 0.01 (0.003 – 0.05) | No change in significance of indirect effects |
| WMI | 0.08 ± 0.16 (-0.23 – 0.39) | -0.12 ± 0.18 (-0.48 – 0.24) | 0.05 ± 0.04 (-0.01 – 0.13) | 0.13 ± 0.06 (0.03 – 0.25) | 0.02 ± 0.01 (0.003 – 0.05) |  |
| ***Model: Child-informant PBP composite score mediated by global EF*** | | | | | |  |
| VPT | 0.42 ± 0.14  (0.15 – 0.70) | 0.29 ± 0.15  (0.002 – 0.57) | 0.02 ± 0.04  (-0.06 – 0.08) | 0.08 ± 0.05  (0.008 – 0.19) | 0.03 ± 0.02  (0.009 – 0.07) | No change in significance of indirect effects |
| WMI | 0.78 ± 0.18  (0.42 – 1.14) | 0.50 ± 0.19  (0.13 – 0.86) | 0.02 ± 0.04 (-0.06 – 0.10) | 0.22 ± 0.08  (0.09 – 0.40) | 0.04 ± 0.02 (0.01 – 0.08) |  |
| ***Model: Child-informant theory of mind mediated by global EF*** | | | | | |  |
| VPT | -0.32 ± 0.13 (-0.58 – -0.07) | -0.17 ± 0.13 (-0.44 – 0.09) | -0.07 ± 0.03 (-0.15 – -0.02) | -0.05 ± 0.03 (-0.14 – -0.001) | -0.02 ± 0.01 (-0.05 – -0.003) | No change in significance of indirect effects |
| WMI | -0.82 ± 0.20 (-1.22 – -0.42) | -0.57 ± 0.20 (-0.96 – -0.18) | -0.08 ± 0.03 (-0.16 – -0.02) | -0.15 ± 0.07 (-0.29 – -0.03) | -0.03 ± 0.01 (-0.06 – -0.004) |  |
| *Note.* IV, independent variable; DV, dependent variable; M1, mediator 1; M2, mediator 2. Standardized estimates ± standard error and 95% confidence intervals shown. Bootstrapping (5000 samples) used to construct confidence intervals for the indirect effects. Group (FT, VPT, WMI) entered as a multi-categorical independent variable using dummy coding. Estimates for WMI and VPT groups are relative to the FT group. All models adjusted for social disadvantage composite and maternal distress index at the 9-10 year follow-up. | | | | | | |

| **Table S11.** Summary of serial mediation analysis testing short-term/working memory (WM) as a mediator between group and Preterm Behavioral Phenotype (PBP) outcomes (*n* = 202) | | | | | | |
| --- | --- | --- | --- | --- | --- | --- |
| **Effects:** | **Total effect** | **Direct effect** | **Indirect effect 1: WM at age 5 years** | **Indirect effect 2: WM at age 9-10 years** | **Indirect effect 3: WM from age 5 to 9-10 years** |  |
|  | *IV → DV* | *IV → DV controlling for M1 and M2* | *IV* *→ M1→ DV* | *IV → M2→ DV* | *IV → M1**→ M2 → DV* |  |
| ***Model: Parent-informant social communication-interaction differences mediated by WM*** | | | | | | **Conclusion** |
| VPT | 0.41 ± 0.13  (0.15 – 0.66) | 0.35 ± 0.14 (0.07 – 0.62) | 0.03 ± 0.03 (-0.01 – 0.11) | 0.03 ± 0.03 (-0.04 – 0.10) | 0.004 ± 0.01 (-0.006 – 0.02) | No mediation |
| WMI | 0.40 ± 0.17 (0.07 – 0.73) | 0.31 ± 0.19 (-0.07 – 0.69) | 0.04 ± 0.03 (-0.02 – 0.12) | 0.05 ± 0.06 (-0.07 – 0.16) | 0.005 ± 0.01 (-0.006 – 0.02) | No mediation |
| ***Model: Child-informant PBP composite score mediated by WM*** | | | | | |  |
| VPT | 0.56 ± 0.13  (0.31 – 0.82) | 0.45 ± 0.14  (0.18 – 0.72) | 0.03 ± 0.03  (-0.02 – 0.10) | 0.08 ± 0.04  (0.01 – 0.16) | 0.01 ± 0.009  (0.001 – 0.03) | Partial mediation |
| WMI | 0.99 ± 0.20  (0.60 – 1.37) | 0.80 ± 0.20  (0.41 – 1.20) | 0.03 ± 0.03 (-0.02 – 0.11) | 0.14 ± 0.06  (0.03 – 0.27) | 0.01 ± 0.01 (0.001 – 0.04) | Partial mediation |
| ***Model: Child-informant theory of mind mediated by WM*** | | | | | |  |
| VPT | -0.50 ± 0.12 (-0.72 – -0.27) | -0.38 ± 0.12 (-0.62 – -0.14) | -0.05 ± 0.03 (-0.13 – -0.001) | -0.05 ± 0.04 (-0.13 – 0.003) | -0.008 ± 0.007 (-0.03 – -0.001) | Partial mediation |
| WMI | -1.05 ± 0.23 (-1.50 – -0.60) | -0.88 ± 0.23 (-1.34 – -0.42) | -0.06 ± 0.03 (-0.16 – -0.002) | -0.10 ± 0.06 (-0.22 – 0.004) | -0.009 ± 0.008 (-0.03 – 0.001) | Partial mediation |
| *Note.* IV, independent variable; DV, dependent variable; M1, mediator 1; M2, mediator 2. Standardized estimates ± standard error and 95% confidence intervals shown. Bootstrapping (5000 samples) used to construct confidence intervals for the indirect effects. Group (FT, VPT, WMI) entered as a multi-categorical independent variable using dummy coding. Estimates for WMI and VPT groups are relative to the FT group. All models adjusted for social disadvantage composite and maternal distress index at the 9-10 year follow-up. | | | | | | |

**FIGURES**


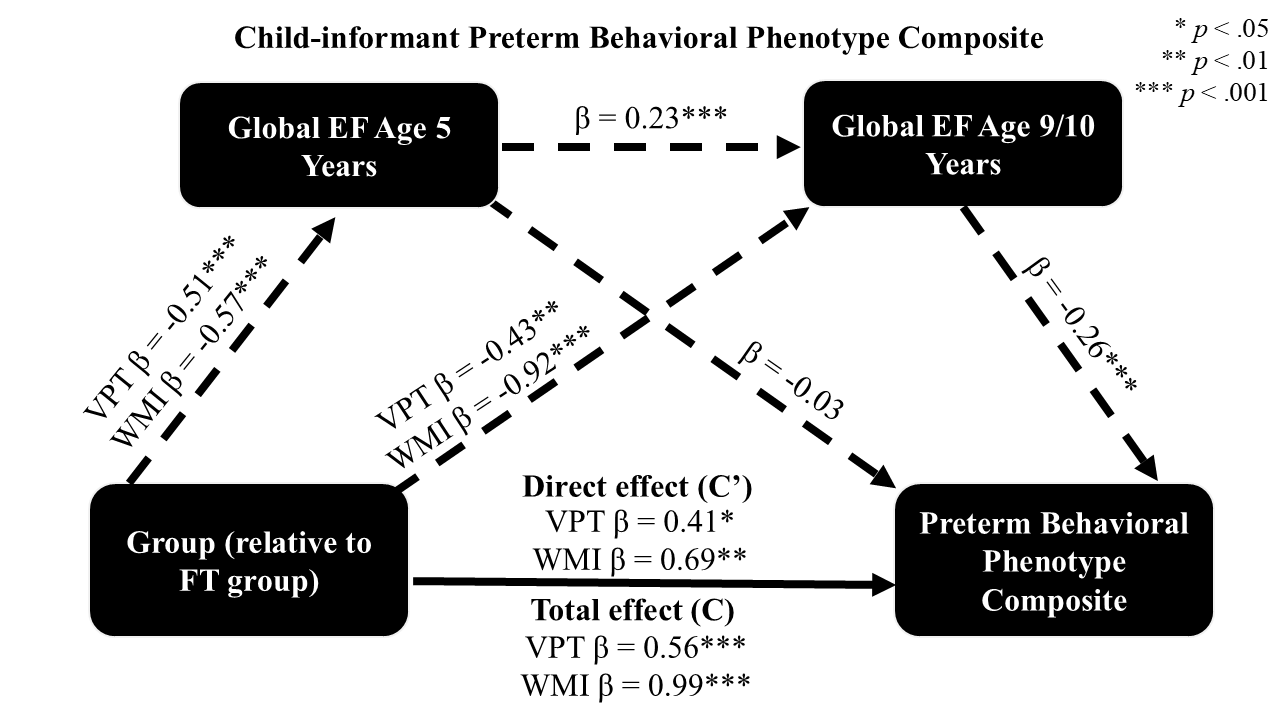


**Figure S1. Serial Mediation Analysis: Path estimates linking group and executive function (EF) with the Preterm Behavioral Phenotype.** Results show that Preterm Behavioral Phenotype outcomes were partially mediated by via two indirect pathways linking group with global EF from age 5 years to age 9-10 years and with EF at age 9-10 years. Model is adjusted for social disadvantage and maternal psychosocial stress at the 9-10 year follow-up.


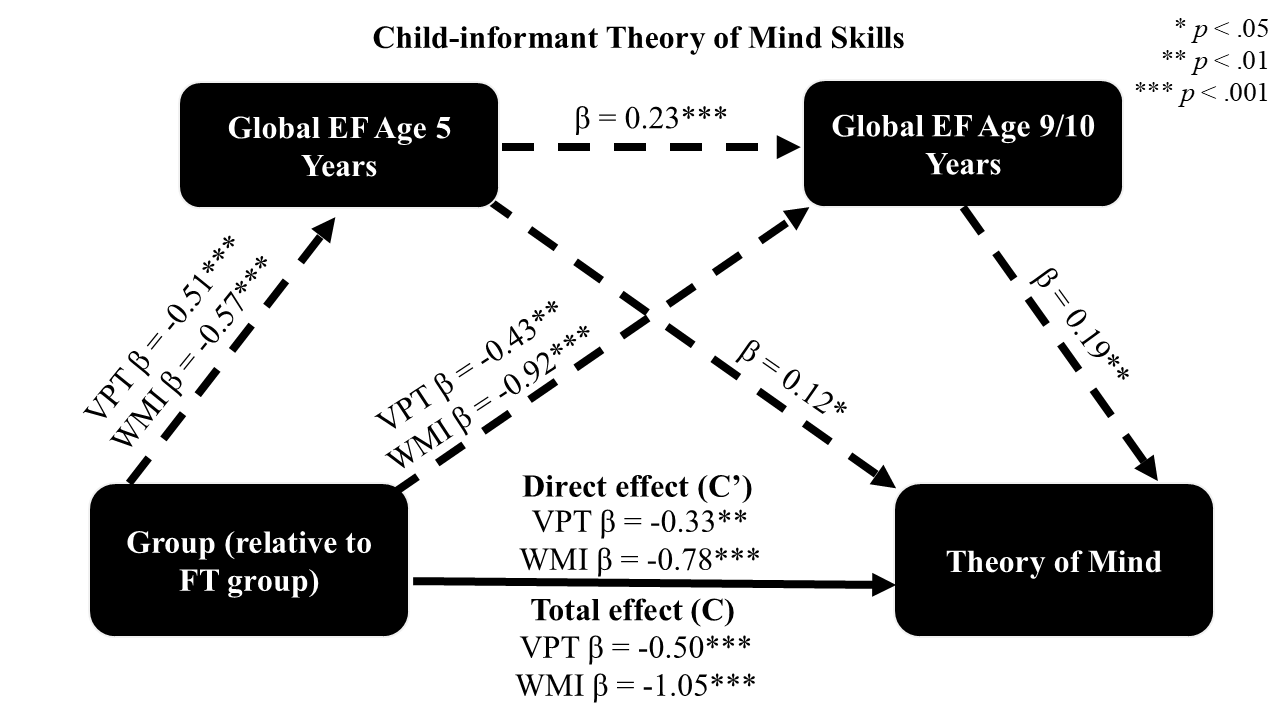


**Figure S2. Serial Mediation Analysis: Path estimates linking group and executive function (EF) with theory of mind skills.** Results show that theory of mind outcomes were partially mediated by via three indirect pathways linking group with global EF at age 5 years, from age 5 years to age 9-10 years, and with EF at age 9-10 years. Model is adjusted for social disadvantage and maternal psychosocial stress at the 9-10 year follow-up.

**Supplementary Material References**

1. Elliott CD. Differential Ability Scales. Second Edition. San Antonio, TX: Harcourt Assessment; 2007.

2. National Institutes of Health Toolbox. Cognition Battery (NIH Toolbox CB). Monogr Soc Res Child Dev. 2013;78(4):1–172.

3. Achenbach T, Ruffle T. The Child Behavior Checklist and related forms for assessing behavioral/emotional problems and competencies. Pediatr Rev. 2000;21(8):265–71.

4. Birmaher B, Khrtarpal S, Brent D, Cully M, Balach L, Kaufam J, et al. The Screen for Child Anxiety Related Emotional Disorders (SCARED): Scale Construction and Psychometric Characteristics. J Am Acad Child Adolesc Psychiatry. 1997;36(4):545–53.

5. Constantino JN, Gruber CP. Social Responsiveness Scale, Second Edition (SRS-2). Los Angeles, CA: Western Psychological Services; 2012.

6. Achenbach TM, McConaughy SH, Ivanova MY, Rescorla LA. Manual for the ASEBA Brief Problem Monitor^TM^ for Ages 6-18 (BPM/6-18). University of Vermont: Resarch Center for Children, Youth, and Families;

7. Brooks BL, Sherman EMS, Strauss E. NEPSY-II: A Developmental Neuropsychological Assessment, Second Edition. Child Neuropsychol. 2009;16(1):80–101.

8. Carter AS, Briggs-Gowan MJ, Jones SM, Little TD. The Infant-Toddler Social and Emotional Assessment (ITSEA): factor structure, reliability, and validity. J Abnorm Child Psychol. 2003 Oct;31(5):495–514.

9. Beck AT, Steer RA, Brown GK. Manual for the Beck Depression Inventory-II. San Antonio, TX: Psychological Corporation; 1996.

10. Spielberger CD, Gorsuch RL, Lushene R, Vagg PR, Jacobs GA. Manual for the State-Trait Anxiety Inventory. Palo Alto, CA: Consulting Psychology Press; 1983.

11. Abidin RR. Parenting Stress Index (PSI). Charlottesville, VA: Pediatric Psychology Stress; 1990.

12. Epstein NB, Baldwin LM, Bishop D. The McMaster family assessment device. J Marital Fam Ther. 1983;9(2):171–80.

13. Belsky J, Crnic K, Gable S. The determinants of coparenting in families with toddler boys: spousal differences and daily hassles. Child Dev. 1995 June;66(3):629–42.

14. Dreyer BP, Mendelsohn AL, Tamis-LeMonda CS. Assessing the Child’s Cognitive Home Environment Through Parental Report; Reliability and Validity. Early Dev Parent. 1996 Dec 1;5(4):271–87.

15. Wechsler D. Wechsler Test of Adult Reading. San Antonio, TX: The Psychological Corporation; 2001.

16. Bishop CL, Lean RE, Smyser TA, Smyser CD, Rogers CE. Adverse Childhood Experiences and Socioemotional Outcomes of Children Born Very Preterm. J Pediatr. 2025 Jan 1;276.

17. Lean RE, Gerstein ED, Smyser TA, Smyser CD, Rogers CE. Socioeconomic disadvantage and parental mood/affective problems links negative parenting and executive dysfunction in children born very preterm. Dev Psychopathol. 2023 Aug;35(3):1092–107.
